# Computational identification and clinical validation of a novel risk signature based on coagulation-related lncRNAs for predicting prognosis, immunotherapy response, and chemosensitivity in colorectal cancer patients

**DOI:** 10.3389/fimmu.2023.1279789

**Published:** 2023-10-19

**Authors:** Fang Zhang, Rixin Zhang, Jinbao Zong, Yufang Hou, Mingxuan Zhou, Zheng Yan, Tiegang Li, Wenqiang Gan, Silin Lv, Liu Yang, Zifan Zeng, Wenyi Zhao, Min Yang

**Affiliations:** ^1^ State Key Laboratory of Bioactive Substances and Function of Natural Medicine, Institute of Materia Medica, Chinese Academy of Medical Sciences and Peking Union Medical College, Beijing, China; ^2^ Clinical Laboratory, The Affiliated Hospital of Qingdao University, Qingdao, China; ^3^ Qingdao Hospital of Traditional Chinese Medicine, The Affiliated Qingdao Hiser Hospital of Qingdao University, Qingdao, China

**Keywords:** colorectal cancer, coagulation, long noncoding RNA, prognostic signature, tumor microenvironment, immunotherapy, chemosensitivity

## Abstract

**Background:**

Coagulation is critically involved in the tumor microenvironment, cancer progression, and prognosis assessment. Nevertheless, the roles of coagulation-related long noncoding RNAs (CRLs) in colorectal cancer (CRC) remain unclear. In this study, an integrated computational framework was constructed to develop a novel coagulation-related lncRNA signature (CRLncSig) to stratify the prognosis of CRC patients, predict response to immunotherapy and chemotherapy in CRC, and explore the potential molecular mechanism.

**Methods:**

CRC samples from The Cancer Genome Atlas (TCGA) were used as the training set, while the substantial bulk or single-cell RNA transcriptomics from Gene Expression Omnibus (GEO) datasets and real-time quantitative PCR (RT-qPCR) data from CRC cell lines and paired frozen tissues were used for validation. We performed unsupervised consensus clustering of CRLs to classify patients into distinct molecular subtypes. We then used stepwise regression to establish the CRLncSig risk model, which stratified patients into high- and low-risk groups. Subsequently, diversified bioinformatics algorithms were used to explore prognosis, biological pathway alteration, immune microenvironment, immunotherapy response, and drug sensitivity across patient subgroups. In addition, weighted gene coexpression network analysis was used to construct an lncRNA–miRNA–mRNA competitive endogenous network. Expression levels of CRLncSig, immune checkpoints, and immunosuppressors were determined using RT-qPCR.

**Results:**

We identified two coagulation subclusters and constructed a risk score model using CRLncSig in CRC, where the patients in cluster 2 and the low-risk group had a better prognosis. The cluster and CRLncSig were confirmed as the independent risk factors, and a CRLncSig-based nomogram exhibited a robust prognostic performance. Notably, the cluster and CRLncSig were identified as the indicators of immune cell infiltration, immunoreactivity phenotype, and immunotherapy efficiency. In addition, we identified a new endogenous network of competing CRLs with microRNA/mRNA, which will provide a foundation for future mechanistic studies of CRLs in the malignant progression of CRC. Moreover, CRLncSig strongly correlated with drug susceptibility.

**Conclusion:**

We developed a reliable CRLncSig to predict the prognosis, immune landscape, immunotherapy response, and drug sensitivity in patients with CRC, which might facilitate optimizing risk stratification, guiding the applications of immunotherapy, and individualized treatments for CRC.

## Introduction

According to the global cancer report released by the International Agency for Research on Cancer of the World Health Organization, colorectal cancer (CRC) has become the third most common and second most lethal cancer in the world, with more than 1.9 million new cases and approximately 900,000 deaths every year (1). With the development of gene profiles, targeted therapy, and immunotherapy, the treatment of advanced CRC has achieved great progress, but the 5-year survival rate of those patients is still less than 15%. Research of molecular mechanisms has confirmed that CRC is not a single type of disease but a group of diseases with highly heterogeneous and complex biological characteristics (2). Differentiating the different subtypes of CRC helps to understand the biological behavior of CRC at the molecular level and provides individualized treatment to patients. Currently, the common staging strategies for CRC are mainly the tumor size, node, and metastasis (TNM) staging system following the American Joint Committee on Cancer (3) standard and the consensus molecular subtype (CMS) system based on gene expression (4). However, there is still a wide variance in the clinical outcome of patients belonging to the same subtype. Immune-checkpoint therapy has revolutionized cancer therapy and become one of the most important anticancer immunotherapy methods. Immune-checkpoint inhibitors (ICIs) can reactivate disabled T cells and block the process of tumor immune escape to inhibit the malignant progression. However, the current benefit of ICIs therapy in CRC is limited to patients with high microsatellite instability, which prevents 85% of CRC patients from benefiting from immunotherapy (5–7). In recent years, with the deepening of the concept of precision medicine, the detection of related biomarkers is an effective supplement for CRC screening and plays an important role in the judgment of individualized prognosis, precision medicine, and efficacy prediction.

There is a certain connection between the occurrence of tumors and the formation of thrombosis. The disorder of the coagulation system often leads to the occurrence of malignant tumors, so patients with malignant tumors are prone to venous thromboembolism (VTE) (8). However, not only can cancer cause VTE, but it may also lead to systemic coagulopathy, thereby leading to disseminated intravascular coagulation or thrombotic microangiopathy (9, 10). Specific expression promoted by tumor cell–associated clots is a unique feature of malignant tumors. The prognostic role of coagulation-related molecules in various cancers has been reported. The models constructed from coagulation-related molecules have good prognostic value in invasive ductal carcinoma and colon cancer (11, 12). A novel platelet-related gene signature has also been developed to predict the prognosis of patients with triple-negative breast cancer (13). In studies related to skin cutaneous melanoma, coagulation molecules could predict patients’ prognosis and tumor microenvironment (TME) (14). It has been shown that the coagulation cascade plays an important role in the TME of hepatocellular carcinoma (HCC). There is a clear correlation between coagulation and TME in HCC, and the coagulation-related risk score can be used as a reliable prognostic biomarker to provide therapeutic benefits for chemotherapy and immunotherapy and help in clinical decision-making for HCC patients (15). In addition, in gastric cancer, the expression level of procoagulant genes is increased, resulting in increased angiogenesis, epithelial–mesenchymal transition (EMT), and TGF-β signaling, thus resulting in a poor prognosis (16). Collectively, the disorder of the coagulation system plays a very important role in the occurrence and development of tumors, and coagulation-related molecules are highly likely to become biomarkers for the prediction of prognosis and treatment in patients with CRC.

Long noncoding RNAs (lncRNAs) are noncoding products with a length of more than 200 nucleotides (17, 18). There is increasing evidence that lncRNAs play roles of oncogenes or antioncogenes in the regulation of tumorigenesis and development (19). However, there are few studies on coagulation-related lncRNAs (CRLs) in cancer. Coagulation Factor XI Antisense RNA 1 (*F11-AS1*) is an lncRNA that has been detected in ovarian cancer and pancreatic cancer (20, 21). It has been found that *F11-AS1* is downregulated in liver hepatocellular carcinoma (LIHC) tissues and cells and can inhibit the proliferation and migration of LIHC cells. *F11-AS1* regulates the expression of *PTEN* through competitive binding with *miR-3146*, thereby inhibiting the progression of LIHC (22). Another CRL, *ncRuPAR* (non-protein coding RNA, upstream of coagulation factor II thrombin receptor [F2R]/protease-activated receptor-1 [PAR-1]), can inhibit tumor cell proliferation and promote apoptosis of human gastric cancer cells by inhibiting PAR-1, PI3K/Akt signaling, and cyclin D1, and its downregulation can promote angiogenesis, invasion, metastasis, and progression (23, 24). However, it is not clear whether CRLs have a regulatory effect on the occurrence and development of CRC. Considering the important role of the coagulation system and lncRNAs in the development of cancer, we assume that CRLs may become a clinically valuable biomarker for CRC.

In this study, we screened the lncRNAs related to the coagulation pathway and successfully established the cluster of CRLs as well as the coagulation-related lncRNA signature (CRLncSig) in CRC. We comprehensively studied the prognostic value, potential mechanism, immune microenvironment, immunotherapy response rate, and drug sensitivity of the cluster and signature constructed using CRLs in CRC and constructed a competing endogenous RNA (ceRNA) network based on CRLs. Our findings suggest that CRLs can be used as novel biomarkers to predict the mortality risk and can elucidate signaling pathways and mechanisms involved in CRC progression. In addition, CRLs can predict the response to immunotherapy and drug sensitivity of CRC patients, which is conducive to the personalized treatment of CRC patients.

## Materials and methods

### Data sources for research

The gene expression profiles and clinical data of 619 CRC tissues and 50 adjacent normal tissues were downloaded from The Cancer Genome Atlas (TCGA, https://www.cancer.gov/tcga). CMSs for the patients in the cohort were downloaded from Colorectal Cancer Subtyping Consortium Synapse (4). We used the annotation file downloaded from GENCODE (https://www.gencodegenes.org) to acquire the gene list of lncRNA. Furthermore, we used the software R to merge the gene list of the lncRNA and the gene expression profiles to obtain the expression of lncRNAs in CRC patients. RNA-sequencing and clinical data for the external validation cohorts GSE147602 (25) and GSE198103 (26), an immunotherapy cohort (GSE181815) (27), and a single-cell RNA-sequencing (scRNA-seq) cohort (GSE136394) (28) were downloaded from the Gene Expression Omnibus (GEO) database (https://www.ncbi.nlm.nih.gov/geo/). The mutation data for TCGA-CRC patients were acquired from TCGA database and analyzed using the package “maftools” (29).

### Identification of CRLs

A total of 454 coagulation-related genes (CRGs) were extracted from the Molecular Signatures Database (MSigDB, https://www.gsea-msigdb.org/gsea/msigdb). We used the Spearman correlation analysis to screen the CRLs. With a threshold of *P-value<* 0.05 and an absolute value of correlation coefficient > 0.3, we confirmed 2680 CRLs.

### Consensus clustering based on CRLs

First, we performed univariate Cox regression analysis in TCGA cohort to obtain CRLs that were related to the prognosis of CRC patients (*P<* 0.05). Then consensus clustering analysis was performed to identify the distinct coagulation patterns based on the expression of 97 prognosis-related CRLs. The univariate Cox regression and consensus clustering analyses were performed using the R packages “survival” and “ConsensusClusterPlus,” respectively.

### Development of a coagulation-related prognostic model

Among the prognosis-related CRLs (*P*< 0.01), a stepwise regression analysis was used for the development of a coagulation-related prognostic model. Afterward, we obtained 10 CRLs (dynein light chain roadblock-type 2 antisense RNA 1 (*DYNLRB2-AS1*), EF-hand calcium binding domain 13 divergent transcript (*EFCAB13-DT*), Ewing sarcoma-associated transcript 1 (*EWSAT1*), long intergenic non-protein coding RNA 645 (*LINC00645*), long intergenic non-protein coding RNA 901 (*LINC00901*), long intergenic non-protein coding RNA 1496 (*LINC01496*), long intergenic non-protein coding RNA 1738 (*LINC01738*), long intergenic non-protein coding RNA 2962 (*LINC02962*), LDL receptor-related protein 1 antisense RNA (*LRP1-AS*), *and* PATJ divergent transcript (*PATJ-DT*) to construct a prognostic model. The risk score of each patient was computed through multivariate Cox regression analysis. Based on the median risk scores, we divided the CRC patients into two groups, namely, the group with a high-risk score and the group with a low-risk score. The stepwise and multivariate Cox regression analyses were performed using the R packages “MASS” and “survival,” respectively. The risk score for each patient was calculated using the following formula:


$$\text{Risk score}=\sum_{i=1}^{10}\text{Expression }(\text{lncRN}{\text{A}}_{\text{i}})\times \text{Coefficient}(\text{lncRN}{\text{A}}_{\text{i}})$$

### Validation of the prognostic value and construction of a nomogram

To evaluate the prognostic value of the clusters and risk scores in TCGA cohort, the Kaplan-Meier (K–M) survival curves were plotted to compare the overall survival (OS), progression-free survival (PFS), or disease-specific survival (DSS) between the clusters and risk groups using the R package “survminer.” To better demonstrate the reliability of our results, we randomly divided the samples in TCGA into training and validation sets at a ratio of 4:6. In addition, to determine the stability of the clusters and risk scores in predicting OS, we analyzed the correlation between the clusters and clinical characteristics and plotted K–M curves of risk scores for different clinical groups.

We constructed a nomogram model to predict the 1-, 3-, 5-, and 7-year OS of TCGA-CRC patients by selecting the risk scores and certain clinical features from the multivariate Cox regression analysis (*P<* 0.05) and tested the predictive ability of this model through calibration and receiver operating characteristic (ROC) curves. The nomogram was constructed using the R packages “survival” and “regplot,” and the calibration and ROC curves were drawn using the R packages “rms” and “timeROC,” respectively.

### Functional enrichment analysis of differentially expressed genes

We used the R package “limma” to mine differentially expressed genes (DEGs) in different clusters and risk groups, with filtering criteria of an adjusted *P*-value< 0.05 and an absolute value of log_2_ (fold change (FC) > 0.5. We performed pathway enrichment analysis of DEGs using the R package “clusterProfiler” (30), including the Kyoto Encyclopedia of Genes and Genomes (KEGG) pathway analysis, Gene Ontology (GO) analysis, and Gene Set Variation Analysis (GSVA). GSVA was performed using the R package “GSVA” (31).

### Immune profile analysis

First, we used the Estimation of STromal and Immune cells in MAlignant Tumours using Expression data (ESTIMATE) algorithm to evaluate the immunity scores of different clusters and risk groups, including estimate, immune, and stromal scores (32). Subsequently, we downloaded the immune cell infiltration data of TCGA-CRC patients from the TIMER 2.0 database (Tumor IMmune Estimation Resource, https://cistrome.shinyapps.io/timer/), including multiple deconvolution algorithms to assess the extent of immune cell infiltration of patients, namely, the Estimating the Proportion of Immune and Cancer cells (EPIC) (33), the Microenvironment Cell Populations-counter (MCP-counter) (34), the quantification of the Tumor Immune contexture from human RNA-seq data (quanTIseq) (35), TIMER (36), and Xcell (37) algorithms. We used the GSVA algorithm to evaluate the score of six immune-related gene sets [hematopoietic cell kinase (HCK), lymphocyte-specific protein tyrosine kinase, immunoglobulin G (IgG), major histocompatibility complex (MHC) I and II, and signal transducer and activator of transcription 1 (STAT1)] in different clusters (38) and compared the expression of different immunomodulatory genes among the clusters and risk groups. The gene list of immunomodulatory genes was downloaded from the TISIDB database (http://cis.hku.hk/TISIDB/download.php) (39).

### Analysis of immunotherapy efficacy

The Tumor Immune Dysfunction and Exclusion (TIDE) algorithm can be used to predict a patient’s response to ICI therapy. The TIDE score is superior to the recognized immunotherapy biomarkers (tumor mutation burden (TMB), PDL1 level, and interferon γ) for measuring the effect of anti-PD1 and anti-CTLA4 treatment (40). The TIDE score, T-cell exclusion score, and the infiltration of cancer-associated fibroblast (CAF) were retrieved from the TIDE portal (http://tide.dfci.harvard.edu) by entering the normalized transcriptome data of TCGA-CRC patients. The Cytolytic activity (CYT) score is a novel immunotherapy biomarker that reflects the antitumor immune activity of CD8+ cytotoxic T cells and macrophages, which can be assessed by calculating the geometric mean of the expression of *GZMA* and *PRF1 (* 41). The T-cell–inflamed gene expression profile (GEP) score is a predictive score of the efficacy of ICIs widely used in preclinical studies. The GEP score was calculated using the 18 characteristic gene sets of IFN-gamma inflammation. Log2 log conversion was performed on the TPM values of each gene, and the average of the 18 genes was calculated (42).

### Construction of a ceRNA network

To construct an lncRNA–miRNA–mRNA competitive endogenous network (ceRNA), we used weighted gene coexpression network analysis (WGCNA) to screen out the CRLs most correlated with the clusters and risk scores (hub-CRLs) and the immune-related CRGs (immune-CRGs) (43). Next, the miRcode database was used to obtain the miRNA family that bound to hub-CRLs (44). To improve the reliability of the results, mRNAs bound to miRNAs were obtained from the TargetScan (45), miRDB (46), and miRTarBase databases (47), and the results of the three databases were intersected with the immune-CRGs obtained previously. Finally, the ceRNA network was visualized using the software Cytoscape (version 3.9.1, U.S. National Institute of General Medical Sciences).

### Drug-sensitivity prediction

To predict the drug sensitivity of TCGA-CRC patients according to the risk scores, the drug information from three databases, namely the Genomics of Drug Sensitivity in Cancer (GDSC) (48), the Cancer Therapeutics Response Portal (CTRP) (49–51), and the Profiling Relative Inhibition Simultaneously in Mixtures (PRISM), was included in our research (52, 53). We also acquired the transcription data of cell lines from DepMap Public 22Q2 (https://depmap.org/portal/download/), and the prediction was made using the R package “oncoPredict” (54).

### scRNA-seq analysis

We used the scRNA-seq cohort GSE136394, which contained single-cell sequencing data of tumor-infiltrating lymphocytes from five CRC patients to perform scRNA-seq analysis. The R packages “Seurat” (55) and “harmony” (56) were used to read sample data and remove batch effects between the samples, and then we used the TSNE method for dimension reduction processing to obtain the clusters. Finally, we performed cell type annotation through Cellmarker 2.0 website (http://bio-bigdata.hrbmu.edu.cn/CellMarker/index.html).

### Cell lines and cell culture

For the validation of our previous results, we used two human CRC cell lines (LOVO cells and HCT116 cells) and a normal human epithelial cell line (HcoEpic cells), which were purchased from BNBIO Company (Beijing, China). All cells were cultured in a constant temperature and humidity cell incubator at 37°C and 5% CO_2_. The corresponding medium for the cells was as follows: LOVO cells [F-12K medium (Gibco, Invitrogen, Paisley, UK)], HCT116 cells [F12/Dulbecco’s modified Eagle medium (Gibco, Invitrogen, Paisley, UK)], and HcoEpic cells [F12/Dulbecco’s modified Eagle medium (Gibco, Invitrogen, Paisley, UK)]. The medium was supplemented with 10% fetal bovine serum (Corning, NY, USA) and 2% penicillin–streptomycin (10,000 units/mL penicillin, 10,000 μg/mL streptomycin; Gibco, Invitrogen, Paisley, UK).

### RNA extraction and real-time quantitative PCR (RT-qPCR) validation

In addition to the three cell types mentioned above, we used samples from 51 patients with CRC. A total of 51 CRC and matched adjacent normal (distance to cancer greater than 5 cm) tissue samples used for RT-qPCR assay were obtained from patients who had been diagnosed with CRC by pathological examination of tissue biopsy and undergone operations at the Affiliated Hospital of Qingdao University. No radiotherapy or chemotherapy was applied before tissue collection. Informed consent was obtained from all of the participating patients. This work was approved by the Research Ethics Committee of The Affiliated Hospital of Qingdao University and was performed following the 1964 Helsinki Declaration and its later revisions. We used an RNeasy kit (Beyotime, Shanghai, China, R0027) following the manufacturer’s instructions to extract RNA from the cells and tissues. Then, we reverse-transcribed 1 μg of total RNA using SuperScript II reverse transcriptase (TaKaRa, Japan, RR047). Quantitative PCR analysis was performed using SYBR Green Master Mix (TaKaRa, Japan, RR820) with an ABI 7900 HT real-time PCR system. The primer sequences for RT-qPCR are listed in **Supplementary Table S1**.

### Statistical analysis

We used the software IBM SPSS Statistics 25, RStudio 4.2, and GraphPad Prism 8 for the analysis and visualization of the data. Differences were analyzed using the two-sided Wilcoxon rank-sum test for non-normally distributed continuous data and the chi-square test for categorical data. The correlation between non-normally distributed variables was analyzed using Spearman correlation analyses. The difference in survival between different groups was calculated using the log-rank test, and *P*-values were corrected using the Benjamini–Hochberg method in functional enrichment analysis. A *P-*value lower than 0.05 was considered statistically significant.

## Results

### Consensus clustering identifies two clusters based on CRLs

**Figure 1:**
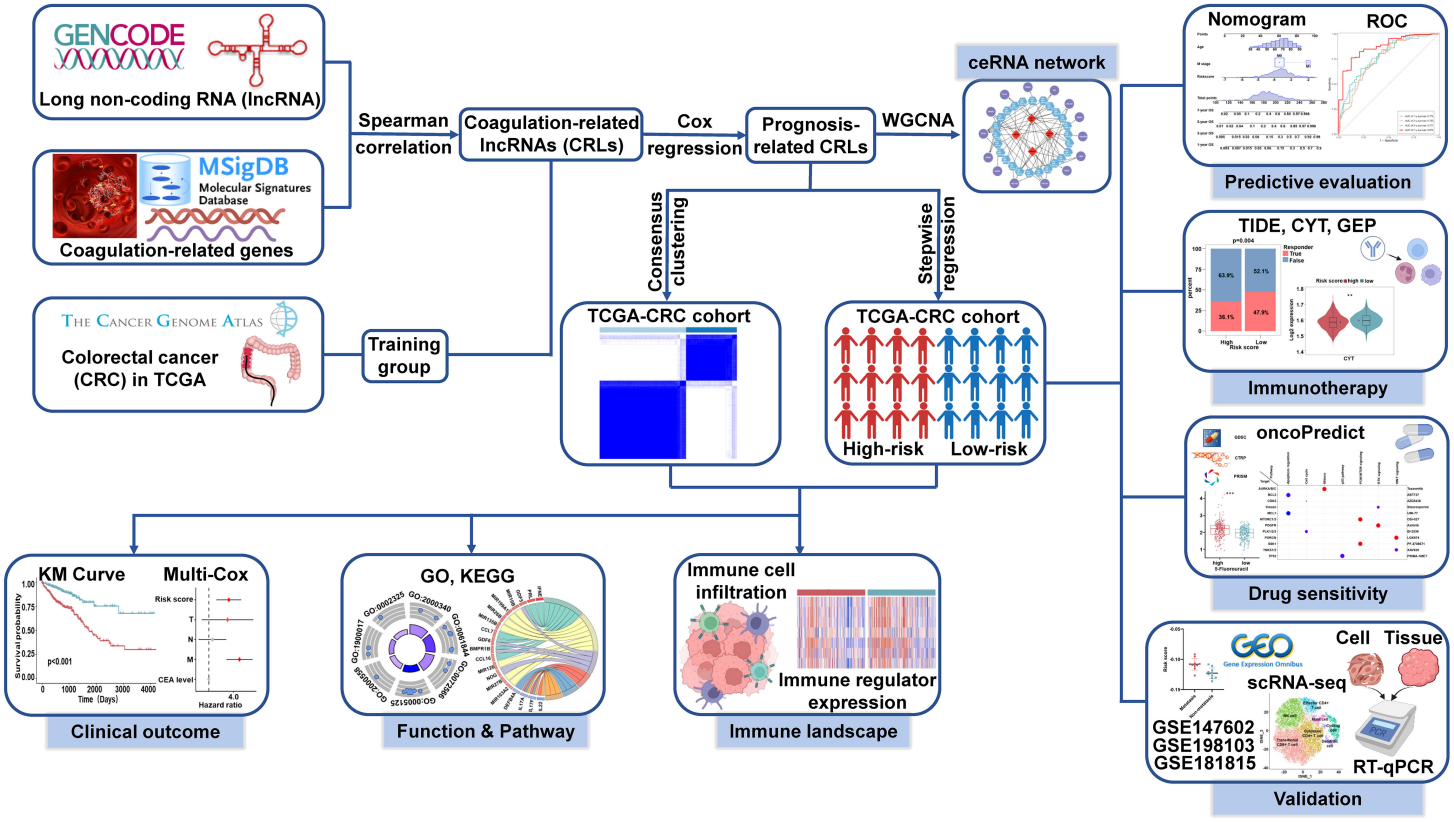
Flowchart of the entire research.

**Figure 2:**
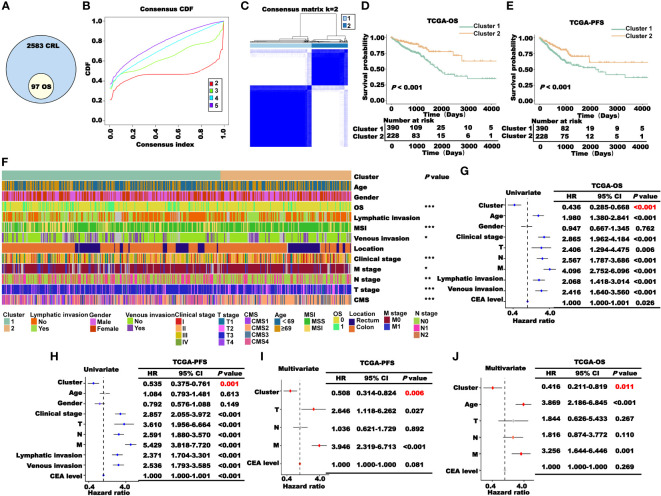
Construction and prognosis analysis of CRL clusters. **(A)** Univariate Cox regression analysis to obtain CRLs associated with prognosis (*P*< 0.05). **(B)** Consensus CDF curve of unsupervised cluster analysis. **(C)** Patients were classified into two clusters through unsupervised cluster analysis. **(D–E)**. **(K–M)** survival curve of two clusters’ OS **(D)** and PFS **(E)** in TCGA cohort. **(F)** Correlation heat map between the cluster and clinical characteristics (*P< 0.05, **P< 0.01, and ***P< 0.001). **(G–J).** Cox regression analysis of the cluster and clinical characteristics. Univariate **(G, H)** and multivariate **(I, J)** regression analyses of TCGA-OS or TCGA-PFS.

The flowchart of the entire research is shown in **Figure 1**. First, we used Spearman correlation analysis of 454 CRGs and obtained a total of 2680 CRLs (*P<* 0.05, |r| > 0.3). Then, we used univariate Cox regression analysis and obtained 97 of the 2680 CRLs associated with prognosis (*P<* 0.05, **Figure 2A**). Subsequently, the 97 CRLs were used for unsupervised cluster analysis. According to the consensus CDF curve, the optimal cluster number was determined to be 2 (**Figure 2B**). After unsupervised clustering, we identified two clusters within TCGA cohort that showed different expression patterns of CRLs (**Figure 2C**). The expression levels of CRLs varied between the different clusters (**Supplementary Figure 1**). Specifically, CRLs with a positive correlation with CRGs were highly expressed in cluster 2, while CRLs with a negative correlation were poorly expressed in cluster 2.

### Prognostic significance of the clusters based on CRLs

According to the results of the K–M survival analysis of OS, PFS, and DSS in TCGA cohort, the patients in cluster 2 had a higher survival probability than those in cluster 1 (**Figures 2D, E**; **Supplementary Figure 2**). Then, we performed a correlation analysis between the clusters and clinical characteristics. As shown in **Figure 2F**, the clusters significantly correlated with OS, MSI, venous invasion, clinical stage, TNM stage, and CMS classification. To explore the prognostic value of the clusters in TCGA patients, Cox regression analysis was performed. First, the clusters and clinical characteristics were incorporated into the univariate Cox regression model, and the *P*-values of the cluster for OS and PFS were lower than 0.05 (**Figures 2G–H**). Subsequently, the factors with *P<* 0.05 in the univariate Cox regression analysis were included in the multivariate Cox regression analysis. As the clinical stage, lymphatic metastasis, and vascular invasion are not independent of the TNM stage, the TNM stage was included in this study. As shown in **Figures 2I, J**, the cluster was a significant independent prognostic factor for CRC.

### Biological pathways and functional enrichment analysis of the clusters based on CRLs

To explore the potential mechanism of the coagulation-related cluster as an independent prognostic factor in CRC, we performed mutation analysis, GO, and KEGG pathway enrichment analysis. Because the occurrence of cancer is closely related to gene mutation, we first performed mutation analysis of clusters 1 and 2 and showed the top 20 mutant genes with a waterfall plot (**Supplementary Figure 3A**). Then, we compared the mutation percentages of 10 common genes closely related to the occurrence of cancer in the two clusters and found that there was no significant difference in the mutation percentages of those genes, except *BRAF* and *RNF43* (**Supplementary Figure 3B**).

**Figure 3:**
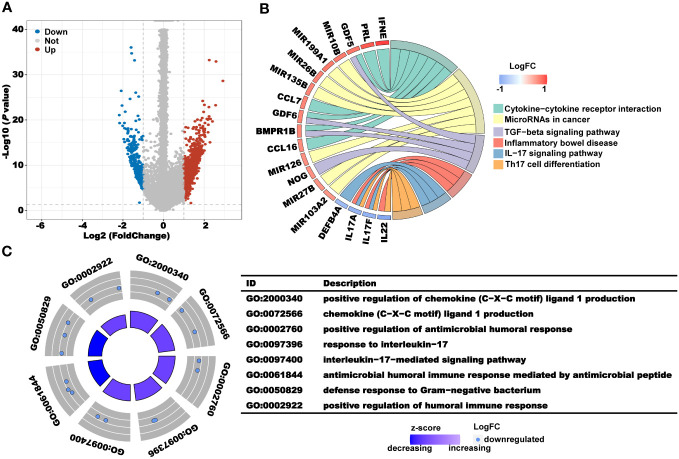
Analysis of the underlying biological pathway of CRL clusters. **(A)** Volcano plot of the differentially expressed genes in clusters 1 and 2 with a threshold of FDR< 0.05 and absolute log2 (FC) > 1. **(B)** KEGG enrichment of differentially expressed genes. **(C)** GO enrichment of differentially expressed genes.

To further explore the potential mechanism, we first used the R package “limma” to mine the DEGs in clusters 1 and 2, and a total of 1624 genes upregulated in cluster 1 and 377 genes upregulated in cluster 2 were obtained (**Figure 3A**). Subsequently, gene name and log_2_FC of the DEGs in the clusters were used for enrichment analysis of GO and KEGG pathways. In the KEGG pathway analysis, the pathways mainly enriched in cluster 1 included cytokine–cytokine receptor interaction, microRNAs in cancer, and the TGF-beta signaling pathway. The pathways mainly enriched in cluster 2 included inflammatory bowel disease, IL-17 signaling pathway, and Th17 cell differentiation (**Figure 3B**). According to the results of GO analysis, immune-related pathways, such as positive regulation of antimicrobial humoral response, response to interleukin-17, and positive regulation of humoral immune response, were mainly enriched in cluster 2 (**Figure 3C**). The above results suggest that the better prognosis of CRC patients in cluster 2 may be due to the anticancer effects of the immune-related pathways.

### Immune profile analysis of the clusters based on CRLs

**Figure 4:**
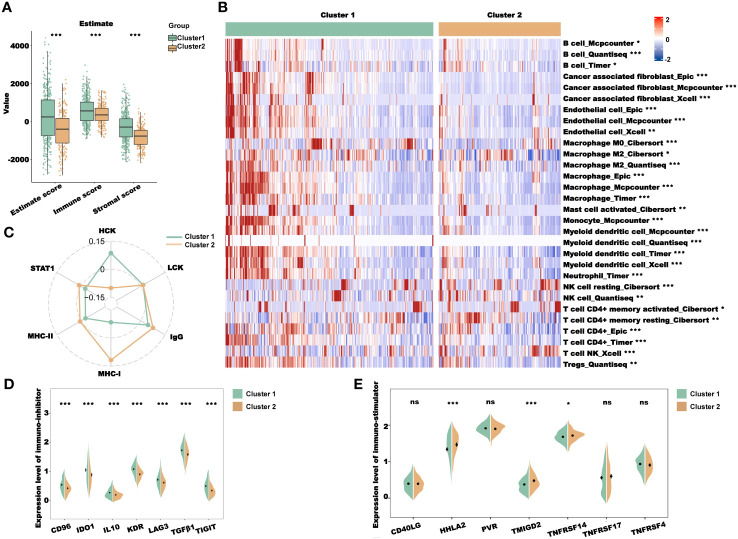
Immune profile analysis of CRL clusters in TCGA-CRC cohort. **(A)** Evaluation of the immune infiltration of clusters 1 and 2 using the ESTIMATE algorithm. **(B)** Evaluation of immune cell infiltration in clusters 1 and 2 using multiple deconvolution algorithms. **(C)** The expression of genes involved in HCK, LCK, IgG, MHC-I, MHC-II, and STAT1 in different clusters. **(D, E)**. Analysis of immune-inhibitor **(D)** and immune-stimulator **(E)** expression in different clusters. HCK, hematopoietic cell kinase; LCK, lymphocyte-specific protein tyrosine kinase; IgG, immunoglobulin G; MHC, major histocompatibility complex; and STAT1, signal transducer and activator of transcription 1. *P< 0.05, **P< 0.01, ***P< 0.001, and ns: no significance.

Previous pathway enrichment results suggest that the prognostic value of the clusters may be related to immunity. In addition, tumor immune microenvironments play a crucial role in the development and progression of tumors. Therefore, we performed the immune profile analysis of the clusters. First, we calculated the estimate, immune, and stromal scores for clusters 1 and 2 using the R package “Estimate.” As shown in **Figure 4A**, the scores of cluster 1 were all higher than those of cluster 2. Subsequently, we downloaded multiple deconvolution algorithms from the TIMER2.0 database to evaluate immune cell infiltration in patients with TCGA-CRC. In cluster 1, CAFs, M2 macrophages, myeloid dendritic cells, and other immunosuppressive cells were infiltrated to a high degree (**Figure 4B**). Furthermore, to investigate the immune system metagene, we examined crucial genes involved in inflammatory activities and immune molecules. We used the GSVA algorithm to calculate the scores of several key gene sets and then compared the scores between clusters 1 and 2. The radar plot presented in **Figure 4C** shows that the HCK score of cluster 1 was significantly higher than that of cluster 2, while the IgG, MHC-I, MHC-II, and STAT1 scores of cluster 2 were significantly higher than those of cluster 1. To further explore the association between the prognostic value of cluster and immunity, we analyzed the differences in the expression levels of immunostimulatory and immunosuppressive genes in clusters 1 and 2. We found that the expression levels of most immunosuppressive genes, such as *CD96*, *IDO1*, *IL10*, and *KDR*, increased in cluster 1 (**Figure 4D**), while the expression levels of some immune stimulatory genes, such as *HHLA2*, *TMIGD2*, and *TNFRSF14*, increased in cluster 2 (**Figure 4E**). These results further indicated that the prognostic value of clusters was related to immune regulation. The higher immune score of cluster 1 may be due to the higher level of immunosuppressive cell infiltration and immunosuppressive gene expression, which presented an immunosuppressive TME and led to a worse prognosis for patients.

### Construction of a prognostic CRLncSig

**Figure 5:**
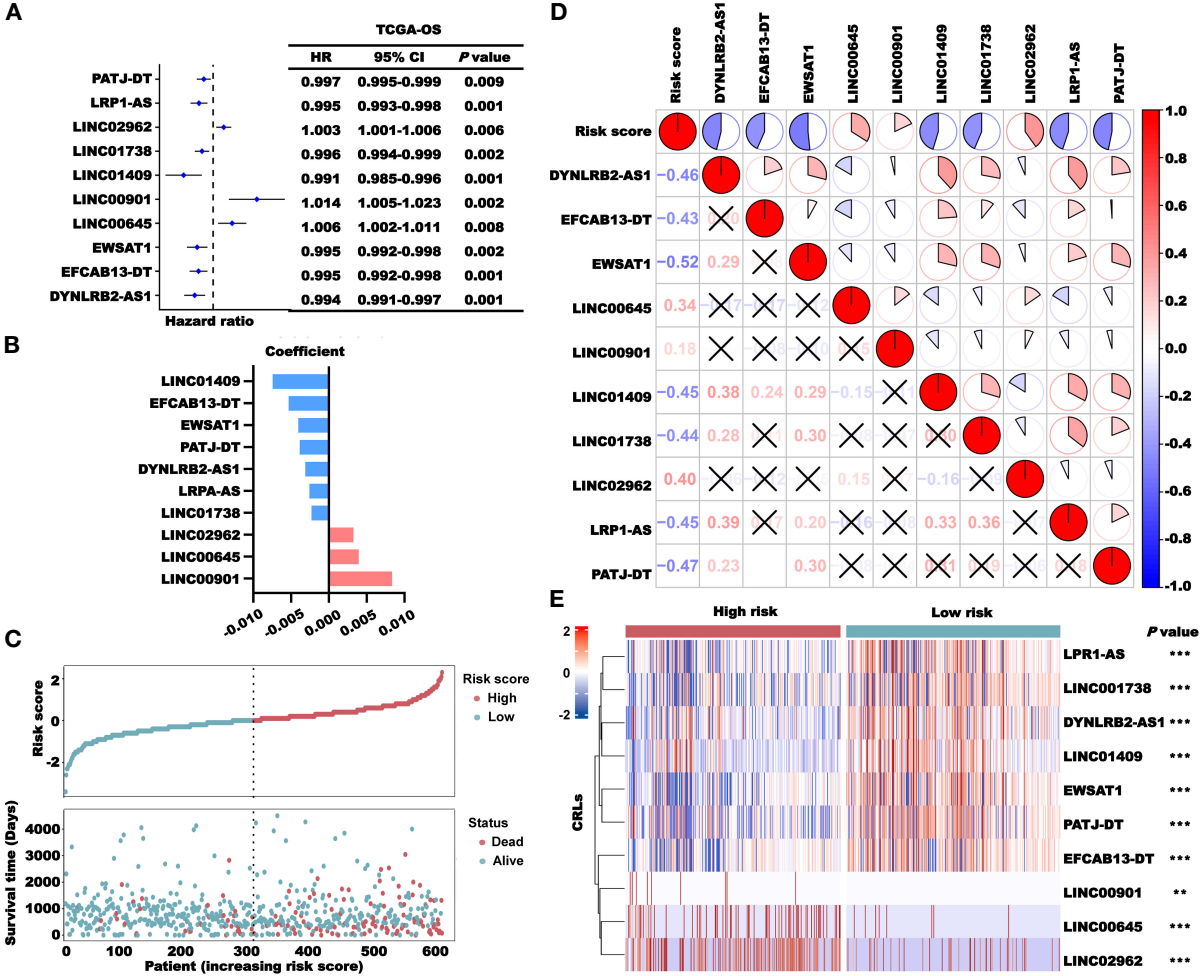
Construction of the CRLncSig. **(A)** Univariate Cox regression analysis of TCGA-OS of the 10 lncRNAs used to construct the signature. **(B)** Coefficients of the 10 lncRNAs in the prognosis signature. **(C)** Distribution of the risk score (high and low) and status (dead and alive) in TCGA-CRC cohort. **(D)** The correlation between the risk score and 10 lncRNAs. **(E)** Expression profiles of the lncRNAs in the high- and low-risk groups. **P< 0.01 and ***P< 0.001.

To construct a prognostic model of CRLs, the aforementioned prognostic CRLs (*P<* 0.01) were used for stepped-regression dimension reduction analysis, and 10 CRLs (*DYNLRB2-AS1*, *EFCAB13-DT*, *EWSAT1*, *LINC00645*, *LINC00901*, *LINC01496*, *LINC01738*, *LINC02962*, *LRP1-AS*, *and PATJ-DT*) were finally obtained for the prognostic model construction. The results of the univariate Cox regression analysis of the 10 CRLs are shown in **Figure 5A**. Next, a multivariate Cox regression analysis was employed to derive regression coefficients for individual genes and compute patient risk scores (**Figure 5B**). **Figure 5C** displays the distribution of risk scores among TCGA-CRC patients. The patients were categorized into high- and low-risk groups based on the median risk score. Subsequently, a correlation analysis was performed to determine the relationship between the risk score and 10 CRLs (**Figure 5D**) and the expression differences of the 10 CRLs between the high- and low-risk groups (**Figure 5E**). The results indicated a high correlation among most of the CRLs. Specifically, the expression levels of *DYNLRB2-AS1, EFCAB13-DT, EWSAT1, LINC01496, LINC01738, LRP1-AS*, and *PATJ-DT* were significantly higher in the high-risk group than in the low-risk group, while the expression levels of *LINC00645, LINC00901*, and *LINC02962* were higher in the low-risk group than in the high-risk group.

### Prognostic significance of the CRLncSig

**Figure 6:**
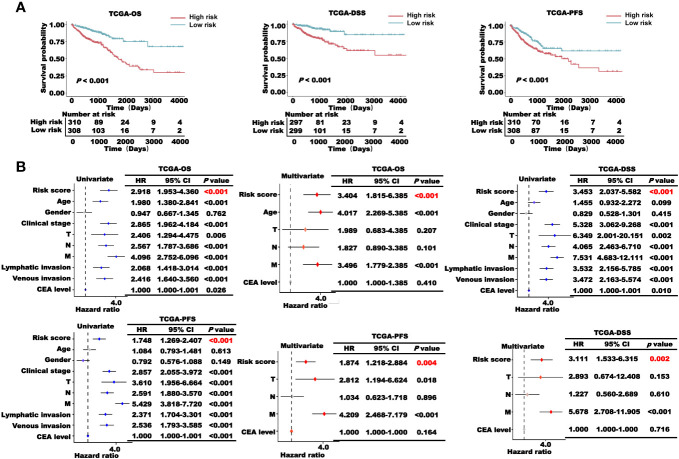
Prognosis analysis of the CRLncSig in TCGA-CRC cohort. **(A)** K–M survival curve of OS, DSS, and PFS of the high- and low-risk groups. **(B)** Univariate and multivariate regression analyses of TCGA-OS, TCGA-PFS, and TCGA-DSS of the risk score and clinical characteristics.

Next, we examined the prognostic role of the risk scores in TCGA-CRC cohort. The results of the K–M survival analysis showed that the patients in the high-risk group had a lower survival probability than those in the low-risk group in terms of OS, DSS, and PFS (**Figure 6A**). In addition, the results of the K-M survival analysis of the training and validation sets showed that patients with high risk scores had worse survival than other patients (**Supplementary Figure 4**). To verify the stability of the model, different clinical characteristics were stratified, and K–M survival analysis was performed independently (**Supplementary Figures 5**, **6**). With different clinical characteristics, the patients in the high-risk group still had a worse survival probability. Subsequently, univariate and multivariate Cox regression analyses were performed to verify whether the risk score was an independent prognostic factor for CRC. The results showed that the *p*-values of the risk score were all lower than 0.05 (**Figure 6B**), indicating that the risk score constructed based on CRLncSig is an independent prognostic factor for CRC.

### Construction and validation of a nomogram combining clinical characteristics

**Figure 7:**
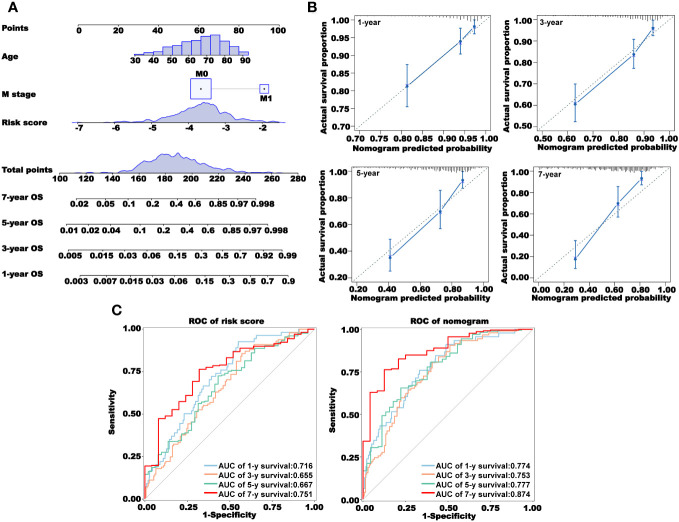
Construction and validation of a nomogram. **(A)** Prediction of 1-, 3-, 5-, and 7-year overall survival using a nomogram constructed using three independent prognostic factors. **(B)** Calibration curves for 1-, 3-, 5-, and 7-year survival. **(C)** Receiver operating characteristic (ROC) curves showing the prediction accuracy of the risk score and nomogram. AUC, area under the curve.

We combined the factors with *P<* 0.05 in TCGA-OS multivariate Cox regression analysis to construct a nomogram model based on the risk score to predict the 1-, 3-, 5-, and 7-year OS of CRC patients (**Figure 7A**). We then used the calibration curve and ROC curve to verify the accuracy of the nomogram model prediction (**Figures 7B, C**). The calibration curve coincided well with the diagonal, and the area under the curve (AUC) values of the 1-, 3-, 5-, and 7-year nomograms were all larger than 0.7, indicating that the nomogram had a good predictive ability.

### Biological pathways and functional enrichment analysis of the CRLncSig

**Figure 8:**
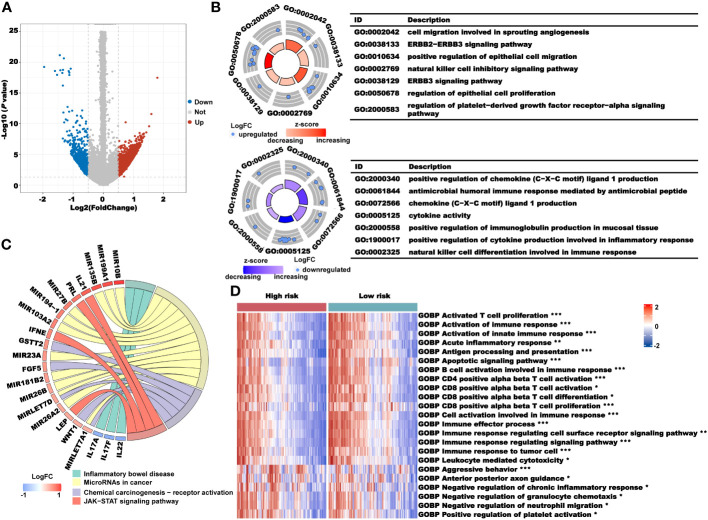
Analysis of the underlying biological pathway of the CRLncSig. **(A)** Volcano plot of the differentially expressed genes in the high- and low-risk groups with a threshold of FDR< 0.05 and absolute log2 (FC) > 0.5. **(B)** GO enrichment of differentially expressed genes. **(C)** KEGG enrichment of differentially expressed genes. **(D)** GSVA enrichment of the high- and low-risk groups. *P< 0.05, **P< 0.01, and ***P< 0.001.

We used the R package “maftool” for the mutation analysis of the high- and low-risk groups (**Supplementary Figure 7**). There was no significant difference in the frequency of the key gene mutations in the process of cancer occurrence between the two groups, indicating that the difference in prognosis between the two groups may not be related to gene mutations. Subsequently, we used the R package “limma” to excavate the DEGs between the high- and low-risk groups. We found that a total of 2840 genes were highly expressed in the high-risk group and 790 genes were highly expressed in the low-risk group (**Figure 8A**). We used these DEGs for GO and KEGG pathway enrichment analyses in the high- and low-risk groups. The results of GO enrichment analysis showed that the main enrichment pathways in the high-risk group were cell migration involved in sprouting angiogenesis, ERBB2–ERBB3 signaling pathway, positive regulation of epithelial cell migration, natural killer cell inhibitory signaling pathway, regulation of epithelial cell proliferation, and regulation of platelet-derived growth factor receptor-alpha signaling pathway. In the low-risk group, the mainly enriched pathways were positive regulation of chemokine (C−X−C motif) ligand 1 production, antimicrobial humoral immune response mediated by antimicrobial peptide, positive regulation of immunoglobulin production in mucosal tissue, positive regulation of cytokine production involved in inflammatory response, and natural killer cell differentiation involved in immune response (**Figure 8B**). Similarly, the KEGG enrichment analysis showed the enrichment of cancer immune-related pathways. microRNAs in cancer, chemical carcinogenesis-receptor activation, chemical carcinogenesis-receptor activation, and JAK−STAT signaling pathway were enriched in the high-risk group, and inflammatory bowel disease was enriched in the low-risk group (**Figure 8C**). From those enrichment results, we speculated that the differential prognosis of the high- and low-risk groups may be mediated through cancer immune pathways. To further verify our conjecture, we performed a GSVA pathway enrichment analysis. As shown in **Figure 8D**, many immune-related pathways were upregulated in the low-risk group, such as activated T-cell proliferation, activation of the immune response, antigen processing and presentation, CD4-positive alpha beta T-cell activation, CD8-positive alpha beta T-cell activation, differentiation and proliferation, and immune response to tumor cells. The pathways that were significantly upregulated in the high-risk group were aggressive behavior, negative regulation of chronic inflammatory response, and positive regulation of platelet activation. These results further illustrated that the prognostic value of our CRL signature in the occurrence and development of CRC was closely related to cancer immunity.

### Immune profile analysis of the CRLncSig

**Figure 9:**
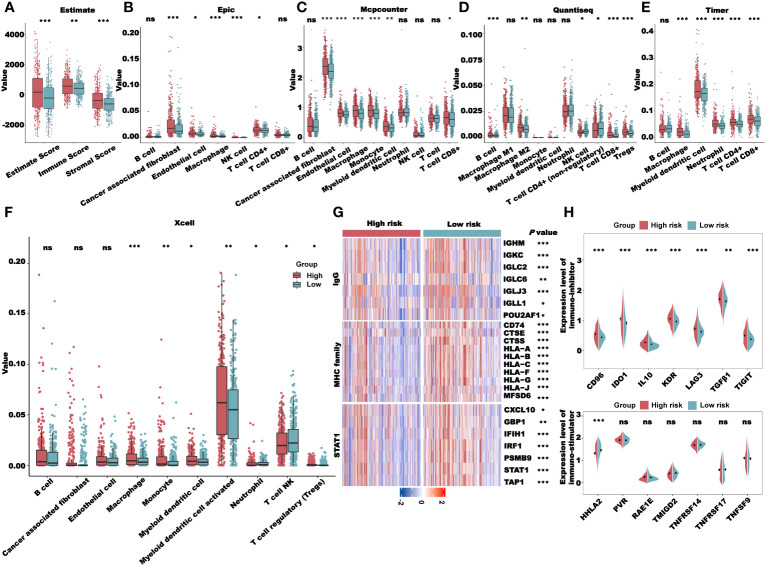
Immune profile analysis of the CRLncSig in TCGA-CRC cohort. **(A)** Evaluation of the immune infiltration of the high- and low-risk groups using the ESTIMATE algorithm. **(B–F)**. Evaluation of immune cell infiltration in the high- and low-risk groups using multiple deconvolution algorithms: EPIC **(B)**, MCP-counter **(C)**, quanTIseq **(D)**, TIMER **(E)**, and Xcell **(F)**. **(G)** The expression of genes involved in IgG, MHC family, and STAT1 in different groups. **(H)** Analysis of immune-inhibitor and immune-stimulator expression in different groups. *P< 0.05, **P< 0.01, ***P< 0.001, and ns: no significance.

Next, we performed an immune profile analysis of the CRLncSig. The results of the Estimate showed that the estimate, immune, and stromal scores of the high-risk group were higher than those of the low-risk group (**Figure 9A**). We used several algorithms, such as EPIC, MCP-counter, quanTIseq, TIMER, and Xcell, to evaluate the degree of immune cell infiltration in the high- and low-risk groups. We found that the immune infiltration of CAFs, M2 macrophages, myeloid dendritic cells, and regulatory T cells in the high-risk group was higher than that in the low-risk group (**Figures 9B–F**). In addition, the expression levels of IgG, MHC family, and STAT1 molecules were higher in the low-risk group than in the high-risk group (**Figure 9G**). However, most of the immunosuppressive genes, such as *CD96*, *IDO1*, *IL10*, *KDR*, *LAG3*, *TGFB1*, and *TIGIT*, were expressed at higher levels in the high-risk group than in the low-risk group, whereas most of the immune stimulatory genes showed no significant differences between the two groups (**Figure 9H**). Therefore, we hypothesized that the patients in the high-risk group had higher immune infiltration and presented an immunosuppressed TME, leading to a worse prognosis.

### Analysis of immunotherapy response based on the CRLncSig

**Figure 10:**
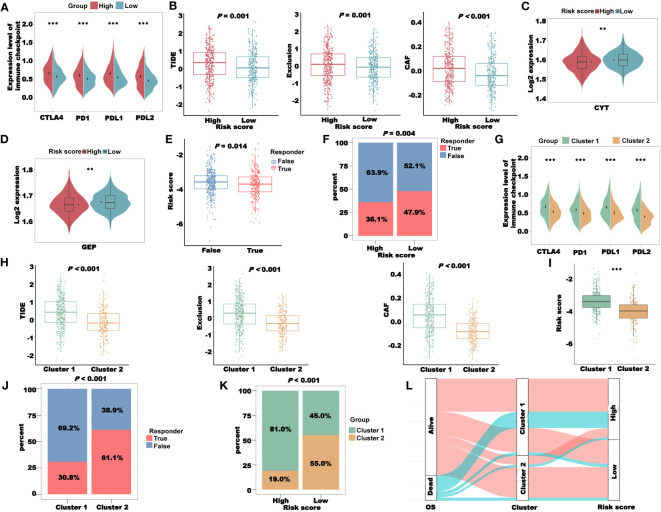
Immunotherapy response analysis of the CRLncSig in TCGA-CRC cohort. **(A)** The expression of key immune checkpoints in the high- and low-risk groups. **(B)** Prediction of patient’s response to immunotherapy in different groups, including TIDE, Exclusion, and CAF scores, using the TIDE algorithm. **(C)** The CYT score of patients in different groups. **(D)** The GEP score of patients in different groups. **(E)** Prediction of the risk score of patients in different responders using the TIDE algorithm. **(F)** The distribution of different responders in high- and low-risk groups. **(G)** The expression of key immune checkpoints in clusters 1 and 2. **(H)** The TIDE, Exclusion, and CAF scores in clusters 1 and 2. **(I)** The difference in risk score between clusters 1 and 2. **(J)** The distribution of different responders in clusters 1 and 2. **(K)** The distribution of clusters in the high- and low-risk groups. **(L)** Sankey diagram combining the OS, Cluster, and risk scores. **P< 0.01 and ***P< 0.001.

From the previous results, we saw a significant correlation between the risk score and the patient’s immune microenvironment. Next, we analyzed the expression levels of several key immune checkpoints in the high- and low-risk groups. As shown in **Figure 10A**, the expression levels of *CTLA4*, *PD1*, *PDL1*, and *PDL2* in the high-risk group were all increased, suggesting that the risk score may be related to the patient’s response to immunotherapy. We used the TIDE algorithm to predict the sensitivity to immunotherapy for TCGA-CRC patients. The TIDE, Exclusion, and CAF scores of the low-risk group were all lower than those of the high-risk group, indicating that the sensitivity of the low-risk group to immunotherapy was higher than that of the high-risk group (**Figure 10B**). We also used the CYT and GEP scores to predict immunotherapy sensitivity in TCGA-CRC patients. Both the CYT and GEP scores of the low-risk group were higher than those of the high-risk group, which further indicated that the sensitivity of the low-risk group to immunotherapy was higher than that of the high-risk group (**Figures 10C–D**). The TIDE algorithm can also predict whether a patient will respond to immunotherapy (False/True). As expected, patients with a False responder had a significantly higher risk score than those with a True responder (**Figure 10E**). Likewise, the proportion of patients with a True responder was significantly higher in the low-risk group than in the high-risk group (**Figure 10F**). Subsequently, we validated the TIDE results using a thymic cancer cohort receiving immunotherapy. The results showed that immunotherapy response rates were lower in patients with high-risk scores and higher in patients with low-risk scores. Then, we used the risk score to predict the immunotherapy response of patients in GSE181815. The results showed that the risk score had an excellent prediction effect (**Supplementary Figure 8**).

To explore the relationship between the coagulation-related clusters and immunotherapy sensitivity, we first compared the expression levels of several key immune checkpoints in clusters 1 and 2 and found that the expression levels of immune checkpoints in cluster 1 were higher than those in cluster 2 (**Figure 10G**). In addition, the TIDE, Exclusion, and CAF scores of cluster 1 were all higher than those of cluster 2, suggesting that cluster 2 patients were more sensitive to immunotherapy (**Figure 10H**). Similarly, in cluster 2, the proportion of patients with a True responder was significantly higher than that of the high-risk group (**Figure 10J**), further indicating that the patients in cluster 2 were more sensitive to immunotherapy. The risk score of cluster 1 was significantly higher than that of cluster 2 (**Figure 10I**), and the proportion of patients belonging to cluster 1 in the high-risk group was significantly higher than the proportion of patients belonging to cluster 2 (**Figure 10K**). These data indicated that our cluster and risk score had a certain correlation. To prove this correlation, we combined the OS, cluster, and risk score of TCGA-CRC patients to draw a Sankey diagram (**Figure 10L**). It was clear that most of the dead patients belonged to cluster 1 and the high-risk group.

### Construction of the ceRNA network based on CRLs

**Figure 11:**
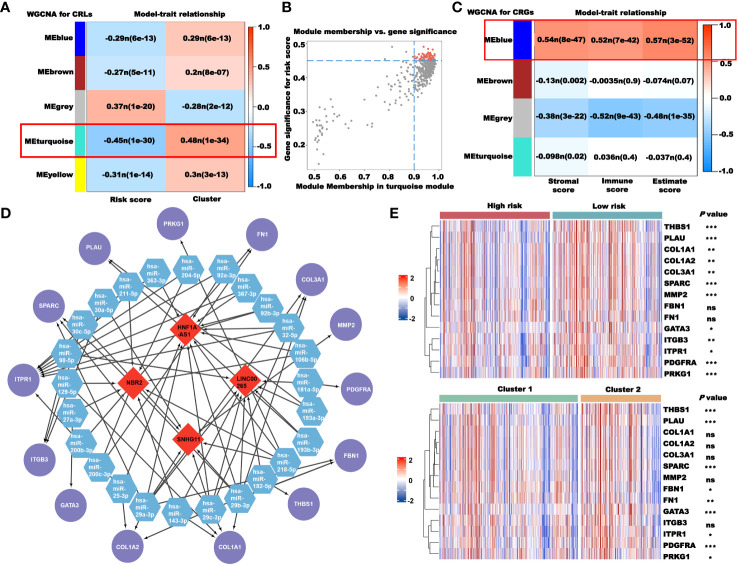
Construction of the ceRNA network based on CRLs. **(A)** Selection of the CRLs that correlated with the risk score and clusters using WGCNA. **(B)** Screening of key CRLs in MEturquoise using correlation scatter map. **(C)** Selection of the CRGs that correlated with immunity using WGCNA. **(D)** The ceRNA network constructed using Cytoscape (red: CRLs; blue: miRNAs; and purple: CRGs). **(E)** The expression of 14 CRGs in different risk groups and clusters. *P< 0.05, **P< 0.01, ***P< 0.001, and ns: no significance.

We used WGCNA to obtain the CRLs that most correlated with the risk score and cluster, and selected the CRLs in the MEturquoise module as the candidate lncRNAs (**Figure 11A**). To further reduce the number of candidate lncRNAs, we screened the CRLs with the highest correlation in the module (**Figure 11B**). The red part is the final candidate CRLs, abbreviated as hub-CRLs. WGCNA was also used to obtain the most CRGs associated with the stromal, immune, and estimate scores. As shown in **Figure 11C**, we chose CRGs in the MEblue as candidate genes, abbreviated as immune-CRGs. Next, we used the miRcode database to obtain the miRNA family binding to hub-CRLs, and then obtained the mRNA binding to miRNA through the TargetScan, miRcode, and miRTarBase databases, and took the intersection with immune-CRGs. Finally, four hub-CRLs, 25 miRNAs, and 14 immune-CRGs were obtained. Subsequently, we drew the ceRNA network using Cytoscape software (**Figure 11D**). We also analyzed the expression of 14 immune-CRGs in different risk groups and clusters, and the expression levels of most genes, including *THBS1*, *PLAU*, *SPARC*, *GATA3*, *ITPR1*, *PDGFRA*, and *PRKG1*, were different among different groups (**Figure 11E**).

### Drug-sensitivity prediction in the CRC patients in the high- and low-risk groups

**Figure 12:**
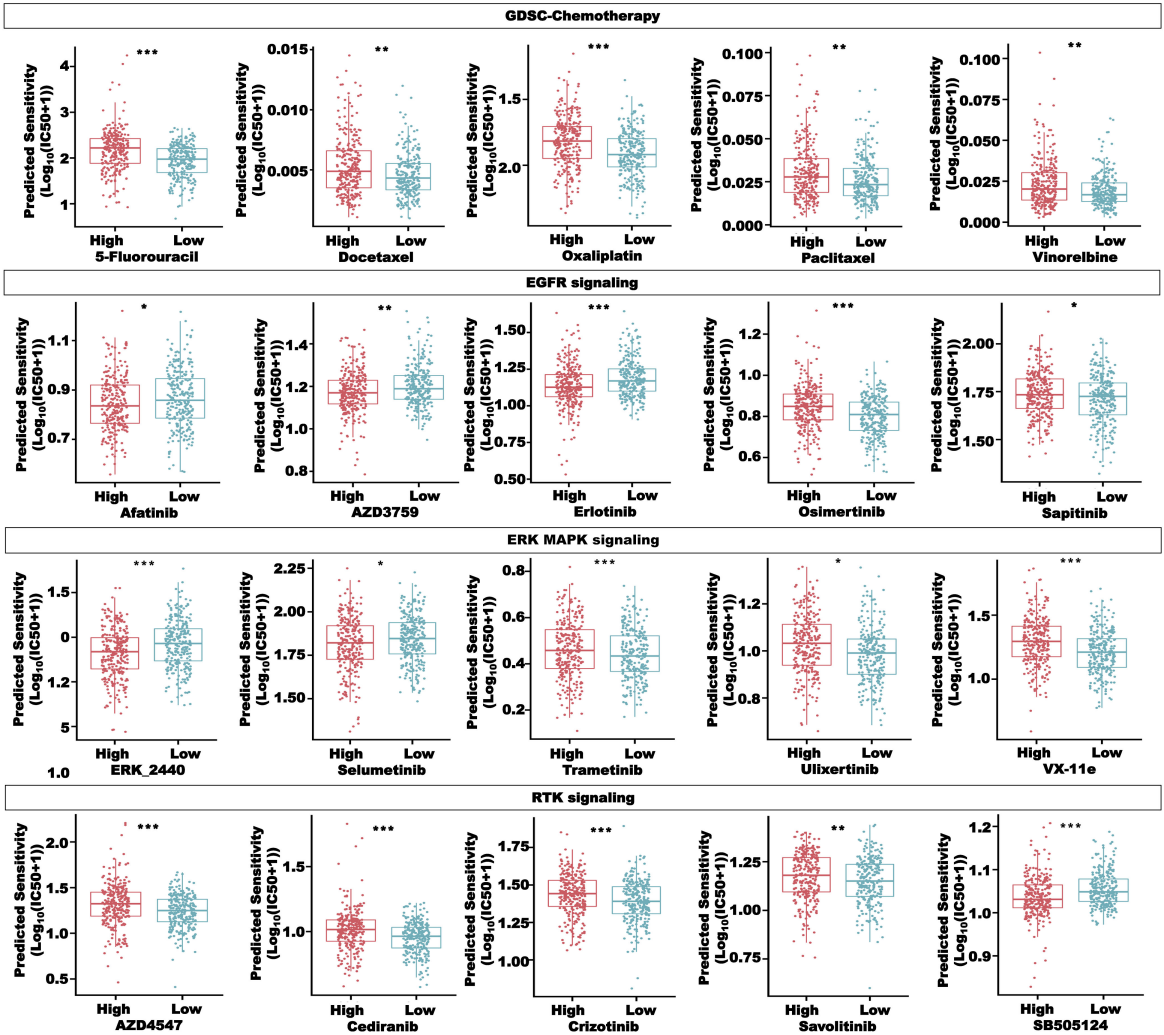
Drug sensitivity analysis in the high- and low-risk groups, including compounds in the GSDC database, targeting chemotherapy, EGFR signaling, ERK MAPK signaling, and RTK signaling. *P< 0.05, **P< 0.01, and ***P< 0.001.

To better apply our CRLncSig to the clinic, we used the R package “oncoPredict” to predict drug sensitivity. The half-maximal inhibitory concentration (IC_50_) in GDSC and the area under the dose–response curve (AUC) in CTRP and PRISM negatively correlated with drug sensitivity. Among 198 compounds in the GDSC database, 135 compounds showed significant differences in the IC_50_ values between the high- and low-risk groups (*P*< 0.05). As shown in **Figure 12**, the patients in the low-risk group were more sensitive to common chemotherapy drugs, including 5-fluorouracil, docetaxel, oxaliplatin, paclitaxel, and vinorelbine. Compounds targeting the EGFR signaling, ERK/MAPK signaling, and RTK signaling, such as afatinib, AZD3759, erlotinib, ERK_2440, selumetinib, and SB505124, were predicted to be more effective in the high-risk group. However, osimertinib, sapitinib, trametinib, ulixertinib, VX-11e, AZD4547, cediranib, crizotinib, and savolitinib were predicted to be more effective in the low-risk group.

**Figure 13:**
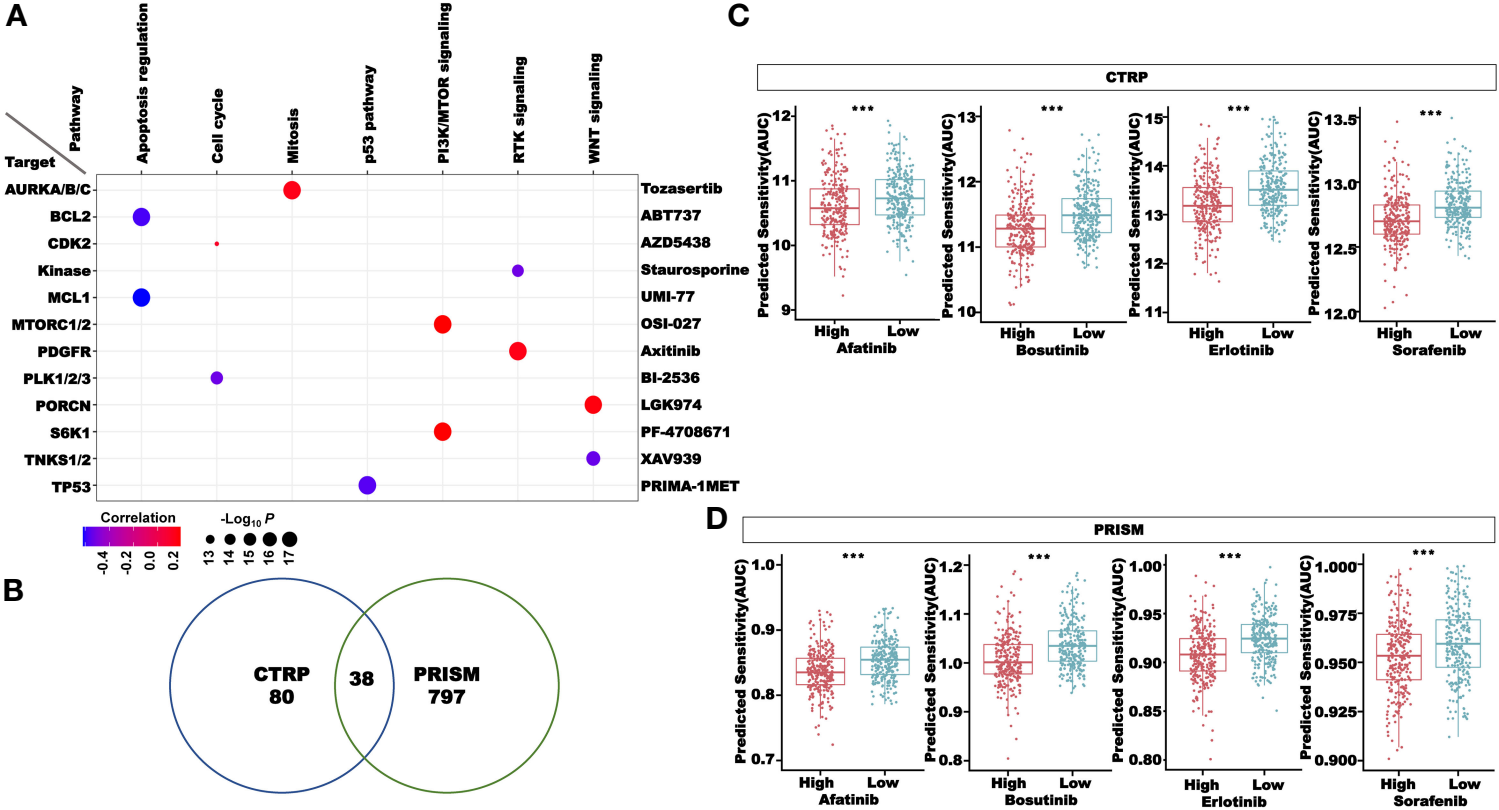
Drug sensitivity analysis in the high- and low-risk groups. **(A)** The correlation between the risk score and compounds in the GDSC database. **(B)** Sensitive compounds for the high-risk group shared by the CTRP and PRISM databases. **(C)** Four compounds sensitive for the high-risk group in the CTRP database. **(D)** Four compounds sensitive for the high-risk group in the PRISM database. ***P< 0.001.

In **Figure 13A**, we selected compounds with a strong correlation with the risk score from the GDSC database to draw bubble plots to show both pathway and target information of the compounds. To screen more drugs suitable for high-risk patients, the intersection of drugs with more sensitivity in the high-risk group in the CTRP and PRISM databases was conducted, and a total of 38 compounds were obtained (**Figure 13B**). **Figures 13C, D** showed some of these compounds, such as afatinib, bosutinib, erlotinib, and sorafenib. The above results proved that our CRLncSig can well predict drug sensitivity in patients of TCGA-CRC cohort and is expected to become a drug guidance strategy for patients with CRC in the future.

### Validation of the CRLncSig

**Figure 14:**
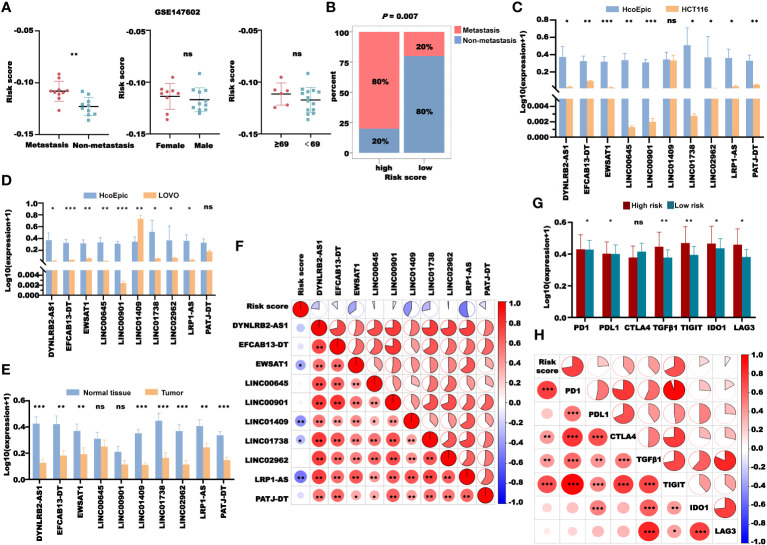
Validation of the CRL signature. **(A)** Risk score distribution in GSE147602 with different clinical features. **(B)** The distribution of patients with or without metastasis in the high- and low-risk groups in GSE147602. **(C)** Comparison of the expression of 10 lncRNAs between HcoEpic cells and HCT116 cells. **(D)** Comparison of the expression of 10 lncRNAs between HcoEpic cells and LOVO cells. **(E)** Comparison of the expression of 10 lncRNAs in normal and tumor clinical samples. **(F)** The correlation between the risk score and 10 lncRNAs in tumor samples. **(G)** The expression of PD1, PDL1, CTLA4, TGFB1, TIGIT, IDO1, and LAG3 in the tumor samples of the high- and low-risk groups. **(H)** The correlation between the risk score and the expression of PD1, PDL1, CTLA4, TGFB1, TIGIT, IDO1, and LAG3 in tumor samples. *P< 0.05, **P< 0.01, and ***P< 0.001, and ns: no significance.

We next validated the clinical significance of our CRLncSig. First, we validated the prognostic value of the risk score in CRC using the GEO dataset GSE147602. The results showed that in GSE147602, CRC patients without metastasis had higher risk scores than those with metastasis, while there were no significant differences in the risk score between patients of different genders and ages (**Figure 14A**). We divided the patients in GSE147602 into the high- and low-risk groups based on the median and found that the proportion of patients without metastasis in the low-risk group was much higher than that with metastasis (**Figure 14B**). Then, we used another GEO dataset, GSE198103, to further verify the ability of this model to predict the prognosis of patients. In GSE198103, the risk scores of patients with a distant metastasis stage were significantly higher than those of patients with a localized stage (**Supplementary Figure 9**). The ability of the coagulation-related lncRNA signature to predict the prognosis of CRC patients was validated with these two cohorts. In addition, we examined the expression of the 10 lncRNAs used to construct the signature in two types of human CRC cells (HCT116 and LOVO) and one type of human normal epithelial cells (HcoEpic) (**Figures 14C, D**). The expression levels of *DYNLRB2-AS1*, *EFCAB13-DT*, *EWSAT1*, *LINC00645*, *LINC00901*, *LINC01738*, *LINC02962*, and *LRP1-AS* in HCT116 and LOVO cells were significantly downregulated compared with those in HcoEpic cells. However, the expression level of *LINC01409* in HCT116 cells was not significantly different from that in HcoEpic cells but was significantly upregulated in LOVO cells. In contrast, there was no significant difference in the expression level of *PATJ* between LOVO and HcoEpic cells, but it was significantly downregulated in HCT116 cells. We also confirmed our previous results in 51 CRC patients. As shown in **Figure 14E**, *DYNLRB2-AS1*, *EFCAB13-DT, EWSAT1*, *LINC01409*, *LINC01738*, *LINC02962*, *LRP1-AS*, *and PATJ-DT* were downregulated in the tumor tissues, while there were no significant differences in the expression level of *LINC00645 and LINC00901* between normal tissue and tumor tissue. We also plotted the correlation graph between the risk score and 10 lncRNAs (**Figure 14F**). In addition, we examined the expression levels of *PD1*, *PDL1*, *CTLA4*, *TGFB1*, *TIGIT*, *IDO1*, and *LAG3* in 51 tumor samples. According to the median, 51 patients were divided into the high- and low-risk groups. We found that the expression levels of *PD1*, *PDL1*, *TGFB1*, *TIGIT*, *IDO1*, and *LAG3* in the high-risk group were significantly higher than those in the low-risk group (**Figure 14G**). The risk score showed a significant negative correlation with the expression levels of *PD1*, *CTLA4*, *TGFB1*, and *TIGIT* (**Figure 14H**), which further proved our previous findings.

### scRNA-seq analysis of the CRLncSig

**Figure 15:**
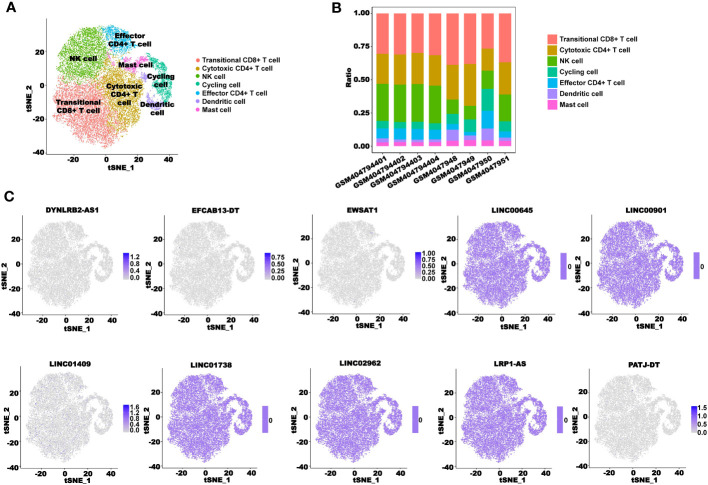
scRNA-seq analysis of the CRLncSig. **(A)** TSNE plot visualization of all cell subtypes from five CRC patients in GSE136394. **(B)** Bar plots of the cell proportions in each CRC patient. **(C)** TSNE plot visualization of the distribution of 10 CRLs.

We used the scRNA-seq cohort GSE136394 to conduct a single-cell analysis of the CRL signature. After TSNE reduction and cell annotation, we obtained seven cell clusters, including transitional CD8+ T cells, cytotoxic CD4+ T cells, NK cells, cycling cells, effector CD4+ T cells, dendritic cells, and mast cells (**Figure 15A**). A total of five samples were included in the GSE136394 dataset, among which four channels were used for single-cell sequencing for the sample GSM4047944. **Figure 15B** shows the proportion of different cells in each sample. Next, we explored the cellular distribution of the 10 lncRNAs used to build the signature. We found that *DYNLRB2-AS1* was mainly distributed in transitional CD8+ T cells, cytotoxic CD4+ T cells, and NK cells. *EFCAB13-DT* was mainly distributed in cytotoxic CD4+ T cells and NK cells, and *EWSAT1* was mainly distributed in transitional CD8+ T cells and effector CD4+ T cells. *LINC01409* was distributed in all of the clustered cells, while *PATJ-DT* was mainly distributed in transitional CD8+ T cells, NK cells, and effector CD4+ T cells. However, the content of *LINC00645*, *LINC00901*, *LINC01738*, *LINC02962*, and *LRP1-AS* in these cells was zero (**Figure 15C**).

## Discussion

Tumor cells can activate the coagulation system and induce a hypercoagulable state in patients with malignant tumors. Previous studies have shown that the incidence of VTE is significantly higher in patients with cancer than in those without cancer (57). It has been reported that the gastrointestinal tract is one of the sites of high incidence of blood coagulation disorders (58, 59). Thromboembolic events are a common cause of death in patients with CRC, even in those with a good cancer prognosis (60). The significant relationship between CRC and thrombosis raises the possibility that the coagulation pathway could be a therapeutic target. lncRNAs are a kind of highly stable noncoding RNAs, and they are also a kind of reliable biomarker of cancer. Currently, the construction of models to predict the prognosis of patients has been proven to be effective and meaningful in many types of cancer (61, 62). In this study, we identified two CRC subtypes based on CRLs and constructed a CRC prognostic signature consisting of 10 CRLs.

The CRLncSig was composed of *DYNLRB2-AS1*, *EFCAB13*, *EWSAT1*, *LINC00645*, *LINC00901*, *LINC01409*, *LINC01738*, *LINC02962*, *LRP1-AS*, and *PATJ-DT*, and six of them (*DYNLRB2-AS1*, *EFCAB13*, *LINC01409*, *LINC01738*, *LRP1-AS*, and *PATJ-DT*) had not been previously reported for their role in tumors. *EWSAT1* has been reported to play a pro-tumor role in a variety of cancers. *EWSAT1* can contribute to the proliferation and invasion of glioma (63), promote HCC metastasis (64), and promote the progression of ovarian cancer (65). Studies on *EWSAT1* in CRC have also been reported. In CRC, the increased expression of *EWSAT1* promotes the proliferation, invasion, and epithelial–mesenchymal transition of CRC cells (66), and also promotes the progression of CRC by regulating *FBXL20* expression through sponging *miR-326 (* 67). Li et al. (68) found that *LINC00645* promoted TGF-β–induced EMT in gliomas by regulating the *miR-205-3p-ZEB1* axis, thereby promoting tumor malignant progression. The study also found that the abnormal expression of *LINC00901* could promote the growth and invasion of pancreatic cancer cells (69). *LINC02962*, also known as *lnc-CCDST*, was found to be significantly downregulated in cervical cancer tissues and bind to pre-carcinogenic DHX9 and E3 ubiquitin ligase MDM2, thereby affecting cervical cancer cell invasion and angiogenesis (70). There are few studies on CRLs in CRC, which has a very good prospect. The signature constructed based on the CRLs also showed excellent clinical value.

Our results indicated that the subtypes and signatures based on CRLs can well distinguish the prognosis of CRC patients. CRC is a highly heterogeneous malignant tumor. The current pathological staging system completely depends on the anatomical degree of the tumor and cannot fully reflect the biological heterogeneity of CRC patients, which affects the accuracy of traditional methods to predict the prognosis of CRC. Herein, we constructed a nomogram model with age, M stage, and risk score to help determine disease progression and personalize diagnosis. The constructed nomogram showed excellent predictive power.

The crosstalk between coagulation and innate immunity is complicated and has been reported in many studies (71, 72). Coagulation is a key component of innate immunity because it prevents the spread of bacteria and can cause inflammation (73). By performing the pathway enrichment analysis, we showed that inflammatory bowel disease and immune-related pathways were enriched in the established signature and clusters. In addition, our immune profile results showed that cluster 1 and the high-risk group were characterized by immunosuppressive microenvironments, in which multiple algorithms indicated a higher proportion of CAFs. CAFs are one of the key components of tumor mesenchyme, which not only provide physical support for epithelial cells but also serve as key functional regulators in cancer. It has been reported that CAFs are associated with poor prognosis and chemotherapy resistance in multiple solid tumors (74, 75). Furthermore, we found that the expression levels of immunosuppressive factors were significantly increased in cluster 1 and the high-risk group, which means that our CRLs in CRC can inhibit the anticancer immune response of patients, thereby leading to the malignant progression.

Interestingly, our study highlighted the potential role of the CRLncSig in predicting the response to immunotherapy in CRC. TIDE, CYT, and GEP scores can predict the response rate of cancer patients to immunotherapy. A greater TIDE score and lower CYT and GEP scores are associated with a greater possibility for immune evasion and exclusion and a lower chance of immunotherapy benefits (40–42). The patients in the high-risk group had higher TIDE scores and lower CYT and GEP scores than the patients in the low-risk group, indicating that the patients in the high-risk group had a lower response rate to immunotherapy. Meanwhile, the TIDE algorithm showed a substantial difference between the nonresponders and responders and predicted a much greater probability of ICI responders among the low-risk patients, explaining why the high-risk patients had a poor prognosis. Additionally, the investigation of drug sensitivity revealed significant differences in the responses to various chemotherapeutic treatments and molecularly targeted medications between the high- and low-risk patients. These results demonstrate that using the risk signature to direct the application of chemotherapy and targeted therapy may be beneficial. As a result, an approach optimizing regimens of a combination of immunotherapy, chemotherapy, and targeted therapy based on the novel CRL signature may be effective for the individualized treatment of patients with CRC.

The ceRNA network is an essential lncRNA-mediated regulatory molecular mechanism that may be used for the investigation of biomarkers, therapeutic targets, and molecular mechanisms (76). In cancer-related studies, most lncRNAs can participate in tumor progression by targeting microRNAs. It has been reported that *lncRNA-CDC6* can promote breast cancer progression by sponging *microRNA-215 (* 77). *lncRNA-RMRP* can promote the proliferation, migration, and invasion of bladder cancer through *miR-206 (* 78). Considering the interactions between lncRNAs and microRNAs, we constructed an lncRNA–miRNA–mRNA ceRNA network to explore the potential regulatory mechanisms of CRLs. Through screening, we obtained four key CRLs, namely, *HNF1A-AS1*, *LINC00265*, *NBR2*, and *SNHG11*. Interestingly, *HNF1A-AS1* can induce resistance to 5-FU in gastric cancer cells via the *miR-30b-5p/EIF5A2* pathway (79). In CRC, *LINC00265* promotes glycolysis and lactate production by regulating the *miR-216b-5p/TRIM44* axis (80), and *NBR2* inhibits cell migration and invasion by downregulating *miRNA-21 (* 81). In addition, *SNHG11* could enhance bevacizumab resistance in CRC by mediating the *miR-1207-5p/ABCC1* axis (82). In this study, we identified a new endogenous network of competing CRLs with microRNA/mRNA, which will provide new ideas for future mechanistic studies of CRLs in the malignant progression of CRC.

In the validation of the CRLncSig at the cellular level, the expression levels of most lncRNAs were decreased in CRC cells (HCT116 and LOVO cells) compared with normal human epithelial cells (HcoEpic cells). These results were verified in CRC and adjacent normal tissue samples. However, we noticed that the expression level of *LINC01409* was significantly higher in LOVO cells than in HcoEpic cells. CRC tissues contained cancer cells and stromal cells such as immune cells, fibroblasts, and blood vessels, which may explain the inconsistencies in RT-qPCR results from our cell and tissue samples. To detect the cell distribution of lncRNAs used to construct the signature in patients with CRC, we performed a single-cell sequencing analysis. From the results, we could see that *LINC01409* was distributed in most immune cells, while other lncRNAs were relatively less distributed in immune cells. Moreover, RT-qPCR was used to detect the expression levels of immune checkpoints and immunosuppressors in CRC tissues, which was consistent with the results in TCGA cohort, further confirming the reliability of our CRL model.

The results showed that our CRLncSig has a good prospect in the precision diagnosis and treatment of CRC. Nonetheless, there are still some limitations to our study. First, most of the current public datasets of cancer-related sequencing fail to contain complete lncRNA expression information; as a result, the number of independent verification sets available is extremely limited. Therefore, only three independent validation sets, GSE147602, GSE198103, and GSE181815, were used to validate our signature in this study, and more independent CRC cohorts, as well as multicenter, large-scale prospective studies, should be integrated to confirm our findings in the future. Second, the mechanisms by which these CRLs affect the immune microenvironment and drug sensitivity of CRC patients are still unclear. Despite our functional enrichment and ceRNA network construction, the biological functions and interactions of these CRLs still need further experimental studies *in vivo* and *in vitro*.

Collectively, this is the most systematic exploration of the clinical significance of CRLs in CRC patients to date. We successfully developed and validated coagulation subtypes and a novel CRL model in CRC. The model performed accurately in predicting the prognosis, immune status, immunotherapy response, and drug sensitivity of CRC patients. This study may provide an innovative perspective for clinical prognosis prediction of patients with CRC and could help deepen the theoretical basis for immunotherapeutic improvement and individualized antitumor therapy.

## Data availability statement

The original contributions presented in the study are included in the article/**Supplementary Material**. Further inquiries can be directed to the corresponding author.

## Ethics statement

The studies involving human participants were reviewed and approved by the Research Ethics Committee of The Affiliated Hospital of Qingdao University. The patients/participants provided their written informed consent to participate in this study. The studies were conducted in accordance with the local legislation and institutional requirements. The participants provided their written informed consent to participate in this study.

## Author contributions

FZ: Formal analysis, Investigation, Methodology, Validation, Visualization, Writing – original draft. RZ: Data curation, Methodology, Writing – original draft. JZ: Data curation, Resources, Writing – original draft. YH: Methodology, Software, Writing – review & editing. MZ: Formal analysis, Validation, Writing – review & editing. ZY: Investigation, Methodology, Writing – review & editing. TL: Software, Visualization, Writing – review & editing. WG: Data curation, Resources, Writing – review & editing. SL: Investigation, Methodology, Writing – review & editing. LY: Validation, Writing – review & editing. ZZ: Data curation, Writing – review & editing. WZ: Formal analysis, Writing – review & editing. MY: Conceptualization, Funding acquisition, Supervision, Writing – review & editing.
