# Supplementary material for: Computational identification and clinical validation of a novel risk signature based on coagulation-related lncRNAs for predicting prognosis, immunotherapy response, and chemosensitivity in colorectal cancer patients
Source: Front Immunol. 2023 Oct 19;14:1279789. doi: 10.3389/fimmu.2023.1279789 (PMC10620970; doi:10.3389/fimmu.2023.1279789)
Supplement: Supplementary file 1 [file DataSheet_1.docx]

**
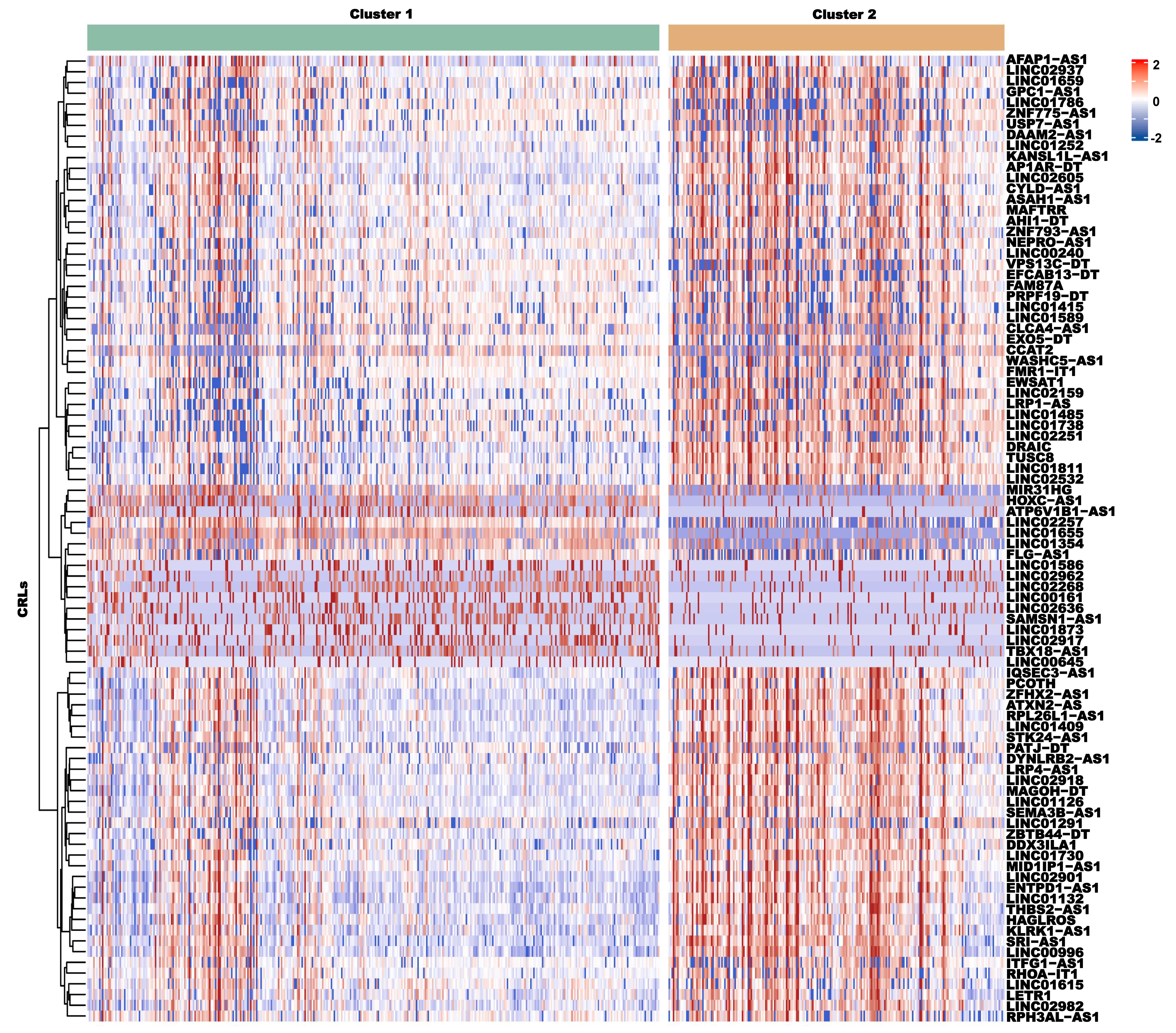
**

**Supplementary Fig. 1.** Distribution of coagulation-related lncRNAs used for unsupervised cluster analysis in clusters 1 and 2 (*P* < 0.05).
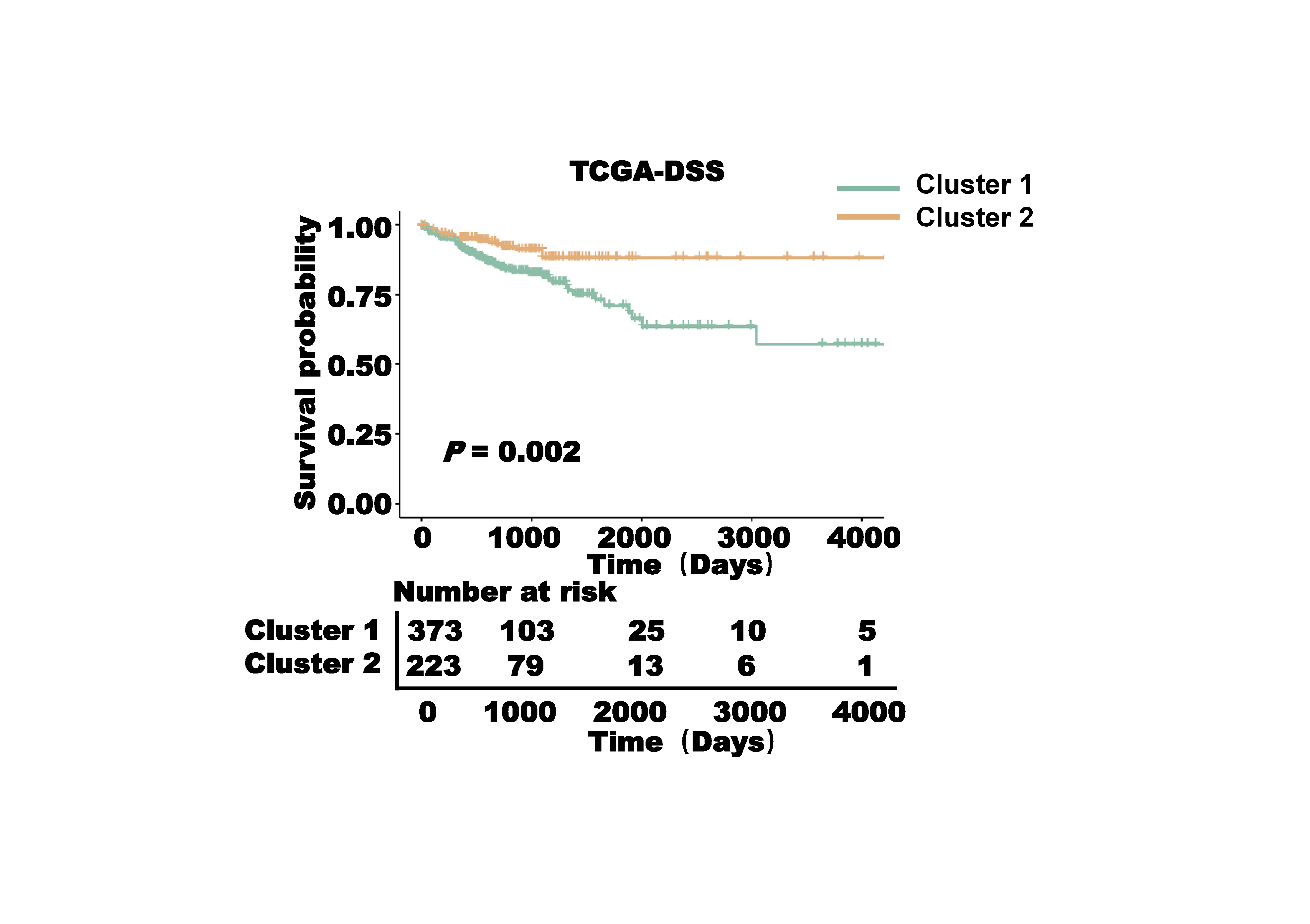


**Supplementary Figure 2.** Difference in DSS between the two clusters.

**
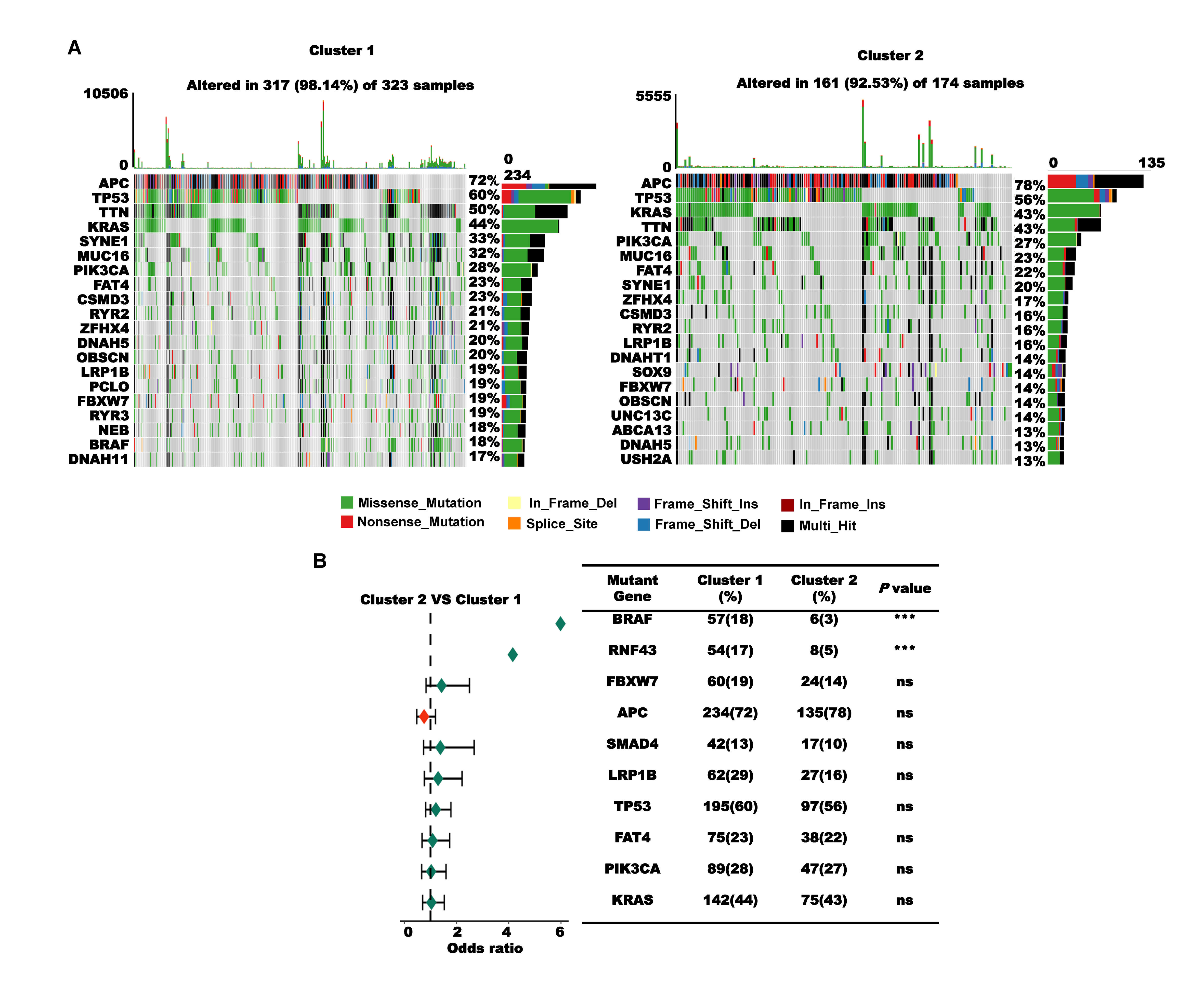
**

**Supplementary Figure 3.** Mutation analysis of clusters 1 and 2. **A**. Top 20 mutated genes in clusters 1 and 2. **B**. Comparison of the mutation ratio of the key genes in clusters 1 and 2.


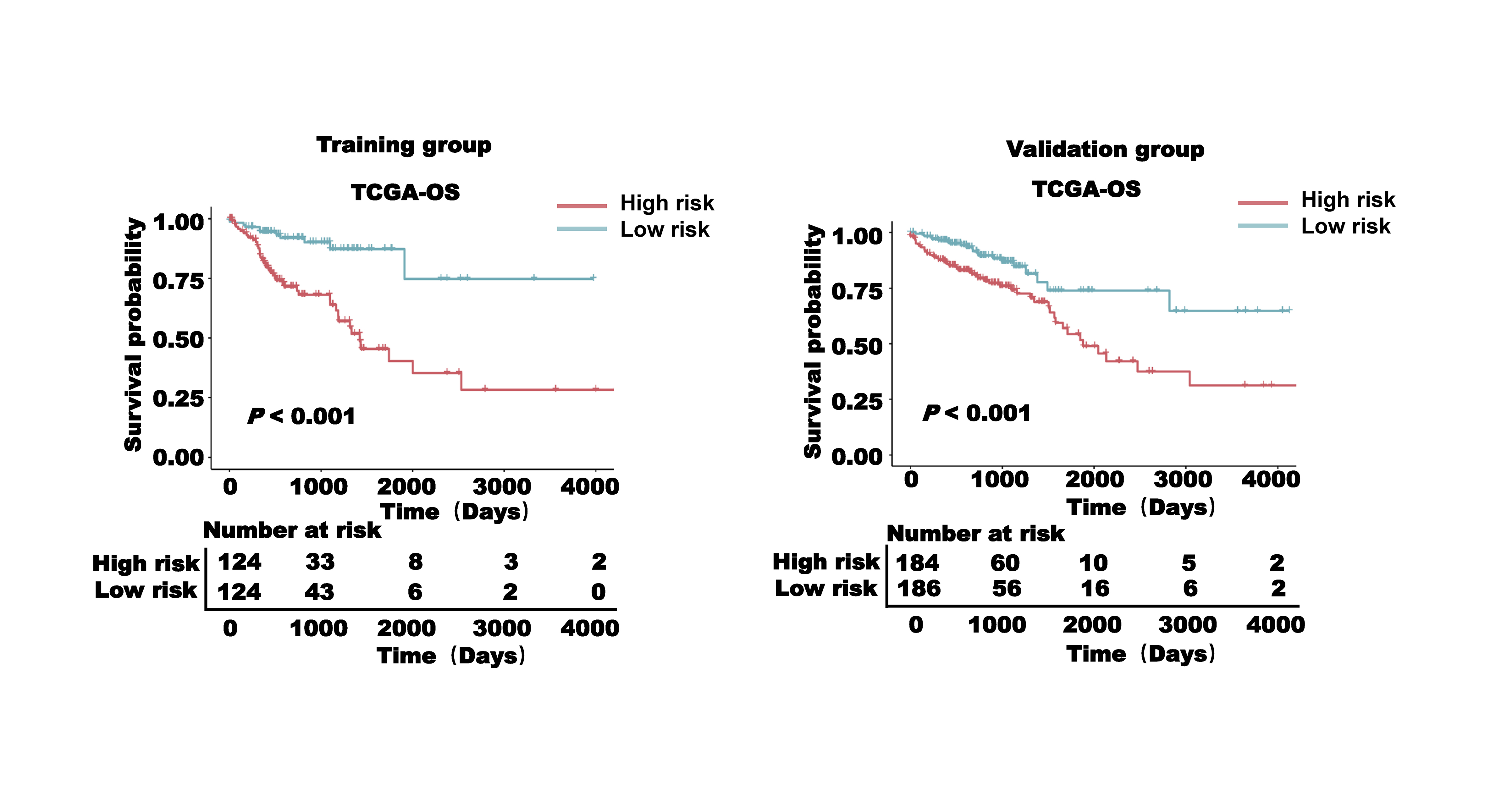


**Supplementary Figure 4.** Survival outcomes of the high- and low-risk groups in the training and validation sets.

**
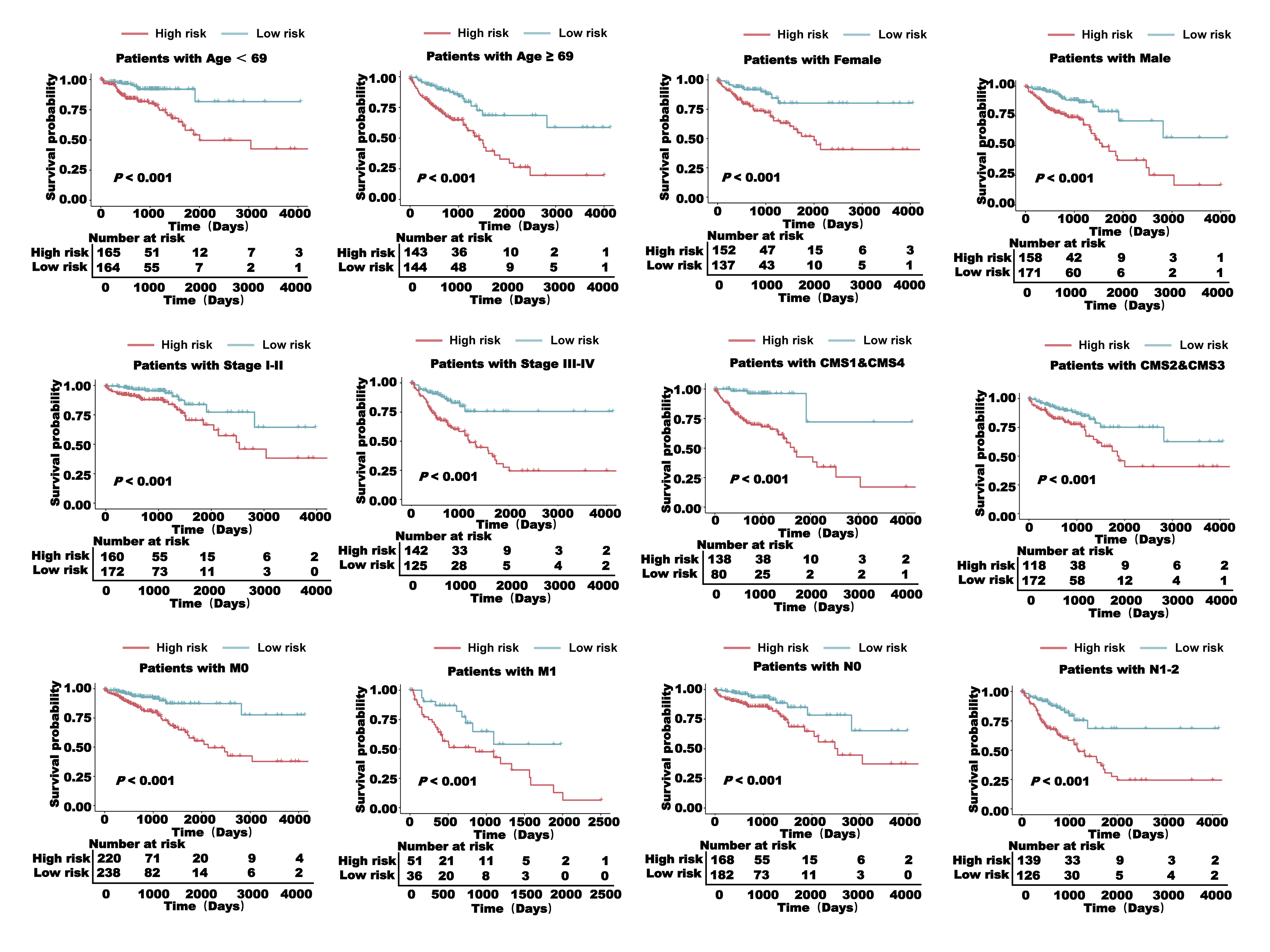
**

**Supplementary Figure 5.** Survival outcomes of the high- and low-risk groups in TCGA-CRC patients were stratified by various clinicopathological features. Kaplan-Meier survival curve showed the survival outcomes of the high- and low-risk groups stratified according to age (≥69 years vs. <69 years), gender (female vs. male), stage (stages I–II vs. III–IV), CMS subtype (CMS1&4 vs. CMS2&3), M stage (M0 vs. M1), and N stage (N0 vs. N1–2).

**
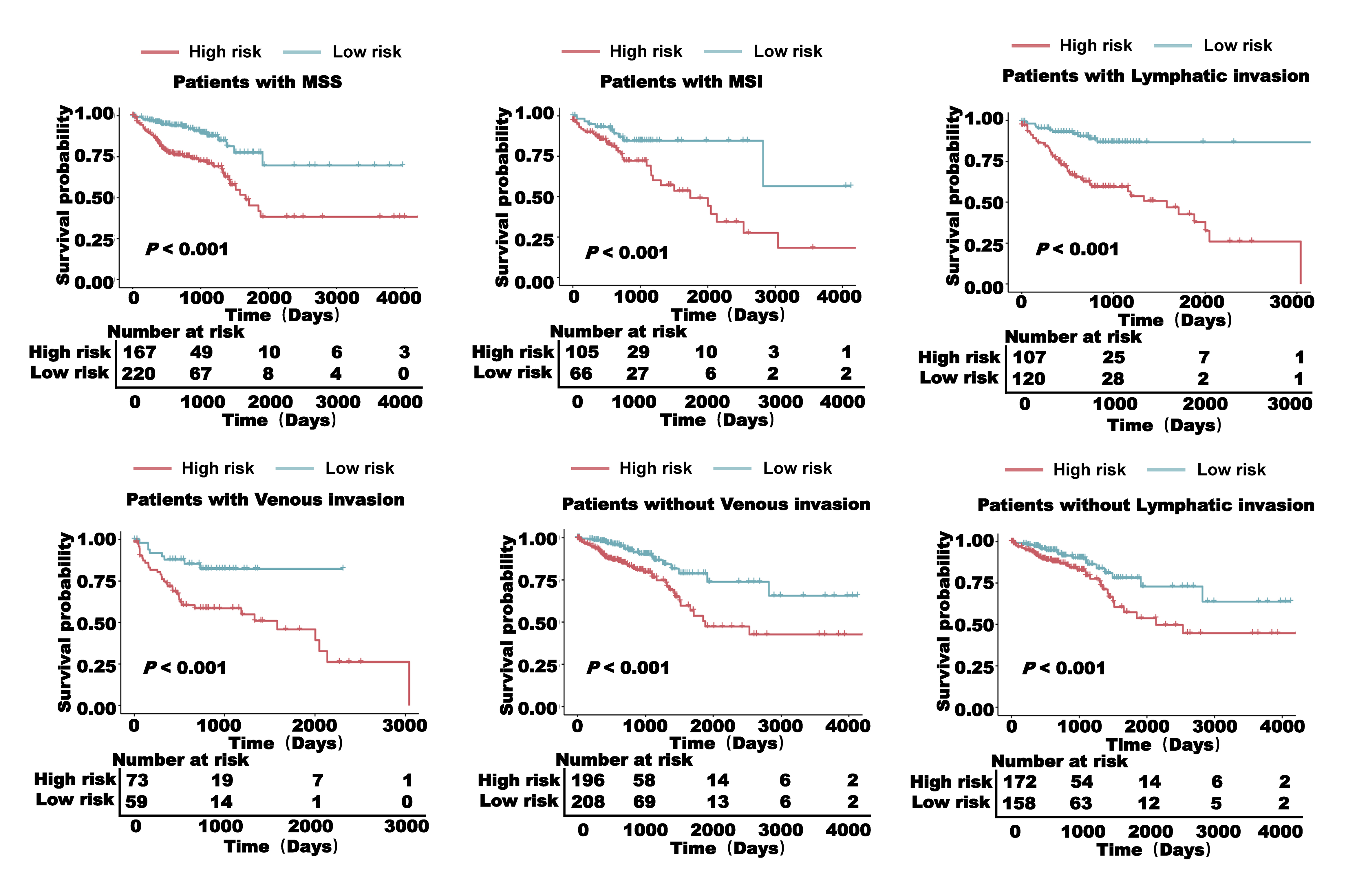
**

**Supplementary Figure 6.** Survival outcomes of the high- and low-risk groups in TCGA-CRC patients were stratified by various clinicopathological features. Kaplan-Meier survival curve showed the survival outcomes of the high- and low-risk groups stratified according to MSI state (MSS vs. MSI), lymphatic invasion (with vs. without), and venous invasion (with vs. without).

**
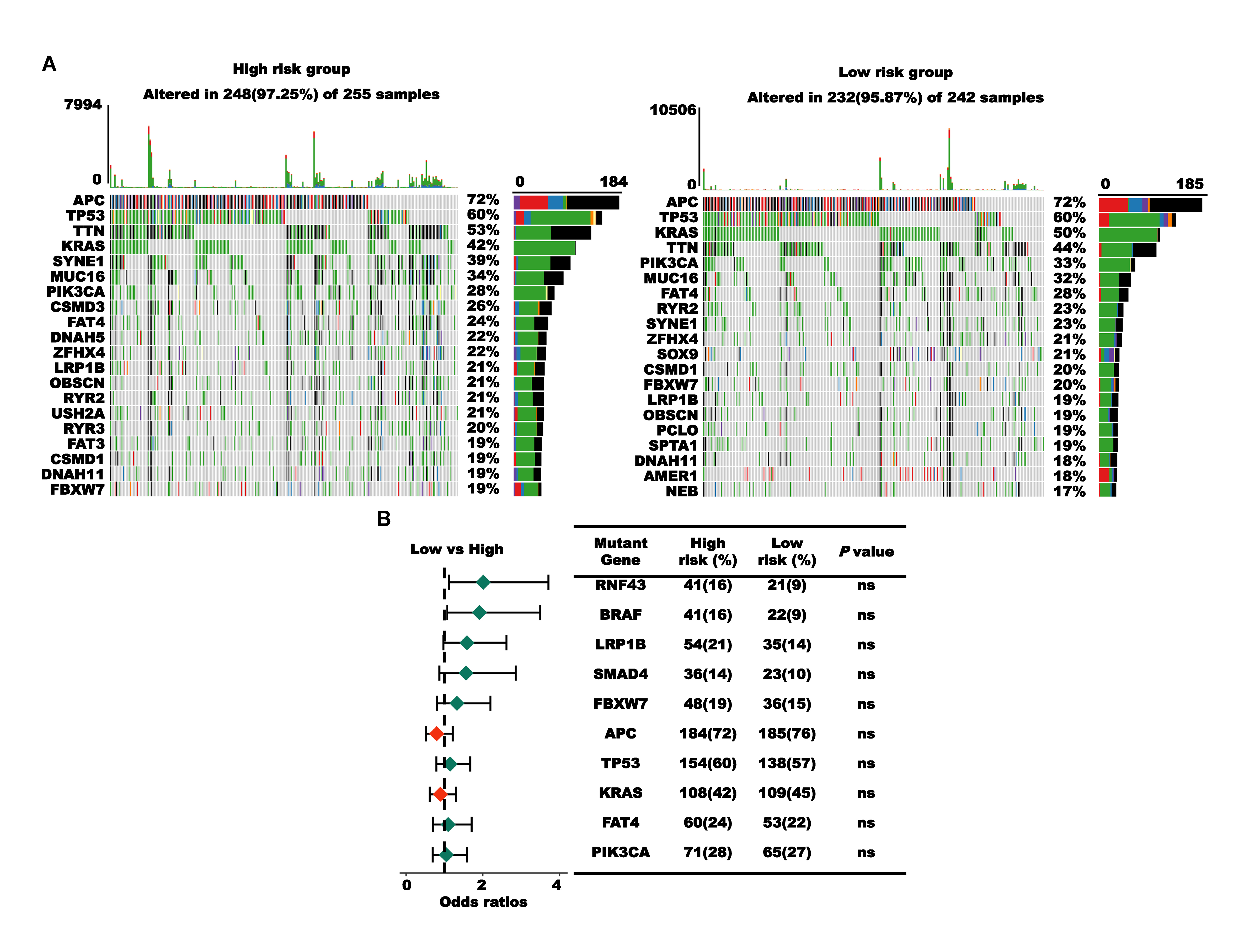
**

**Supplementary Figure 7.** Mutation analysis of the high- and low-risk groups. **A**. Top 20 mutated genes in the high- and low-risk groups; **B**. Comparison of the mutation ratio of the key genes in the high- and low-risk groups.

**
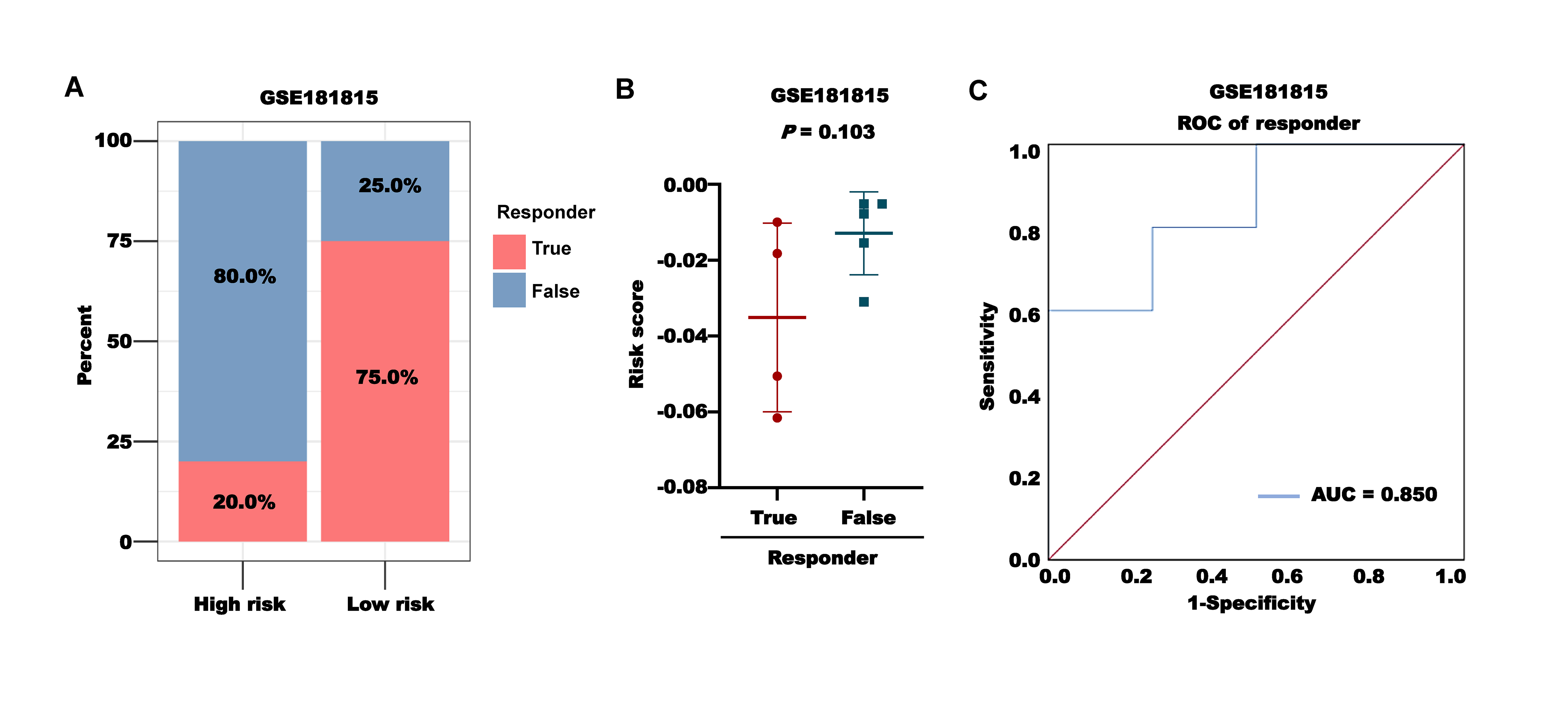
**

**Supplementary Figure 8.** External validation of immunotherapy. **A**. Distribution of different responders in the high- and low-risk groups in GSE181815. **B**. Risk score distribution in GSE181815 with different responders. **C**. ROC curves showing the accuracy of the risk score in predicting the response rate to immunotherapy in GSE181515.

**
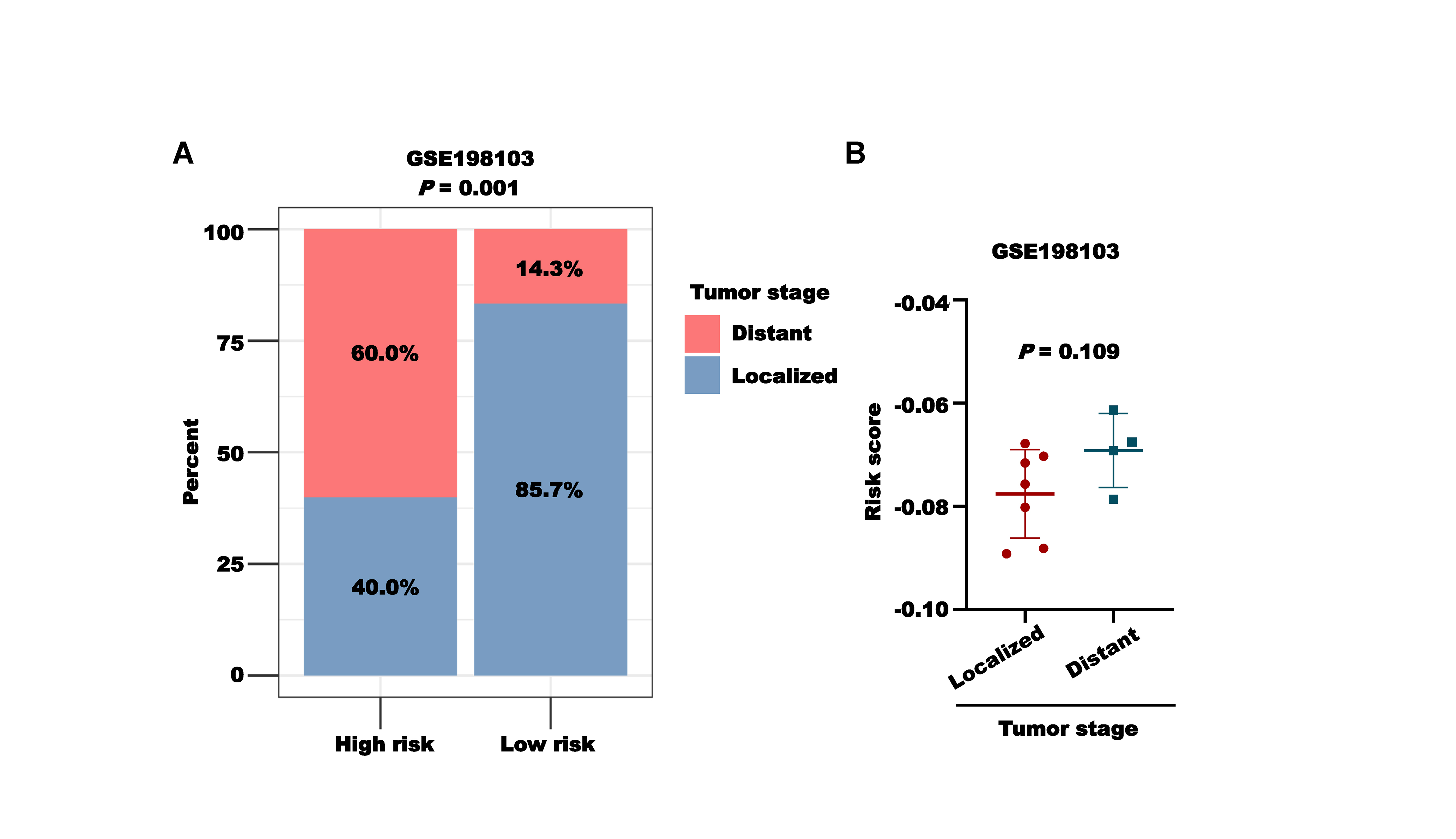
**

**Supplementary Figure 9.** Validation of the prognostic value of the CRLncSig in GSE198103.

**Supplementary Table S1** Primers used for RT-qPCR.

| Name of gene | Sequence (5’→3’) |
| --- | --- |
| DYNLRB2-AS1 F | CTGTAAGGAGCCTGGACATGG |
| DYNLRB2-AS1 R | CCAAAGCGGTCAGAAGAAGTG |
| EFCAB13-DT F | GGGATAAGACTTTGGGGCCT |
| EFCAB13-DT R | TCGATCGCTTGCTTCCCTTT |
| EWSAT1 F | CTGAAACTCCCACCGAGACC |
| EWSAT1 R | ATGGGCTTAAGGGTGGGGTA |
| LINC00645 F | AGCAGCTGTTAAGGTCACTCC |
| LINC00645 R | TGCACAGTTCCATCACCAGA |
| GAPDH-H F | CTGACTTCAACAGCGACACC |
| GAPDH-H R | TGAGCTTGACAAAGTGGTCGT |
| LINC00901 F | GGGGAGCCTGTGTATATGCG |
| LINC00901 R | CAGGTTATCTTCCTGCCGCT |
| LINC01409 F | CCCATCCTGCCACTAACAGA |
| LINC01409 R | GGAGGAAGCCCTTGGTAGAAG |
| LINC01738 F | AGGCCAGCAAAGTCTTGTCA |
| LINC01738 R | TGTCTGAACGTGGCCTTCTC |
| LINC02962 F | GCAGTTCCTGATACTGGTTTTTCT |
| LINC02962 R | CAGCCATGCAATATTCATAGACTTC |
| LRP1-AS F | CGTCTGTGTTGGTGCATAGC |
| LRP1-AS R | CACTTCGTTGCCCTTGTCAC |
| PATJ-DT F | CTGCGTGTAGAGCGAGACC |
| PATJ-DT R | GTTAAGCGGATTGACCCAACG |
| CD274 F | GGAAATTCCGGCAGTGTACC |
| CD274 R | TGACAGCTGGTGGCATTCAA |
| PDCD1 F | CCCAAGGCGCAGATCAA |
| PDCD1 R | GCACTTCTGCCCTTCTCTCTGT |
| CTLA4 F | ACGGGACTCTACATCTGCAAGG |
| CTLA4 R | GGAGGAAGTCAGAATCTGGGCA |
| HLA-A F | TGTGCTCCCTCTCCAATCATC |
| HLA-A R | AGTGCACCATGAAGTTGAGACA |
| HLA-B F | GAATGTGTCTGCGTCCCTGTTA |
| HLA-B R | GAGGAAACACAGGTCAGCATGG |
| HLA-C F | AAATTCATGGTGCACTGAGCTG |
| HLA-C R | ATCCATCAACGCTTCATAGCA |
| TGFB1 F | TGCCCATCGTCTACTACGTG |
| TGFB1 R | TTGCAGGAGCGCACAATCAT |
| TIGIT F | CCTCGCCTCAGGAATGATGA |
| TIGIT R | GTGGTGGAGGAGAGGTGACA |
| IDO1 F | TACCCATTGTAACAGAGCCACA |
| IDO1 R | ATGCACAGGTATTTTGAGGTCTT |

*Note.* F indicates forward; R indicates reverse.
